# Researches into the Physical History of Mankind

**Published:** 1847-10

**Authors:** 


### Art. XIV.

1. *Researches into the Physical History of Mankind.* By James Cowles Prichard, m.d., f.r.s., m.r.i.a., Corresponding Member of the National Institute of France, &c. &c. Third Edition.—*London,* 1836-47. Five Volumes, 8vo, pp. 2547. With numerous Plates.
2. *The Natural History of Man; comprising Inquiries into the Modifying Influence of Physical and Moral Agencies on the different Tribes of the Human Family.* By James Cowles Prichard, m.d., f.r.s., m.r.i.a., Corresponding Member of the National Institute of France, &c. &c. Second Edition.—*London,* 1845. 8vo, pp. 614. With 49 Plates and 97 Wood Engravings.

In the article under this heading in our last Number, we entered into a general examination of the question, whether the differences at present observable between the several races of men are such as to justify the idea that these races have had diverse origins; or whether they are such as present themselves among other races of animals, whose constitutions and habits (like those of man) are capable of adaptation to a great variety of external conditions. From the evidence adduced by Dr. Prichard and others on this question, we felt ourselves warranted in the conclusion that there is no such fixity in the physical characters of particular races of men, as would be required by the zoologist for their separation as distinct species; the characters in which they differ being such as are known by the analogy of other animals to be very liable to variation; and a sufficient amount of testimony as to their variability in man being derivable from actual observation. Moreover it was asserted that a careful examination of all the tribes included under any one designation,—e. g. the African race, the Malay race, or the American race,—would be quite sufficient to dispel the belief that they agree among themselves and differ from all others in any one character or set of characters, such as would be required for the definition of species; since each of these races includes forms that differ more from each other, than they do from some of those included under other races. And it was also stated that a conformity in external conditions and mode of life among people of different races, is so closely associated (where these influences have been sufficiently long in operation) with conformity in certain physical characters, as to warrant the opinion that the latter is mainly dependent on the former. If this be admitted to be the case, it must be obvious that the notion of the origin of these races in five or any number of pairs, differing from each other in the same manner and degree as the European, the Mongolian, the Negro, &c., now differ, must at once fall to the ground; and that we have no resource but to suppose, either that all mankind originated from one pair, or that if the original parents were more numerous they must have had similar physical and mental constitutions.

In their practical bearing upon the estimation in which we are to regard the races that seem at present most degraded below our own level, we do not see that there is any essential difference between these two latter hypotheses. And we cannot think that the last of them is at once to be set aside as inconsistent with the Scriptural narrative; since the liberal interpretation of primeval history, which geological researches have shown to be necessary, might almost as well admit the existence of numerous Adams, as coincide with the now generally-received doctrine of well-informed and unprejudiced thinkers, that the Noachian deluge was very limited in its extent, and did not affect by any means the whole either of the men or animals existing on the earth at the time of its occurrence. We are not aware that anything like demonstrative proof of either of these hypotheses can be obtained by scientific investigations. All analogical probability, however, is in favour of a single centre or original pair; since to such centres, as Dr. Prichard shows in his first volume, the naturalist now refers the origin of all his species of plants and animals. And although there would appear, at a certain stage of the investigation, to be greater facility in accounting for the dispersion of mankind from several stocks than from a single one, yet we believe that the more extensively and profoundly the inquiry is carried on, the more will there be found that *fundamental accordance,* in the midst of great diversity of aspect, which indicates original unity. For ourselves we may say that we commenced the examination of the mass of details which Dr. Prichard has brought together, with a prepossession in favour of numerous centres of radiation; but that we have been gradually led to the belief (upon scientific grounds alone) in the probable existence of but a single original pair.

With these preliminary observations, we shall proceed to give our readers such account of the characters of the principal tribes of mankind, as may enable them to form an opinion for themselves; and we shall commence, as Dr. Prichard has done, with a survey of the African nations, which fills the second volume of his Physical History.

The northern region of the African continent to the westward of Tunis, vaguely termed Mount Atlas, is an elevated country, mountainous in some parts, stretching from the great Sandy Desert to the shores of the Mediterranean and Atlantic. Notwithstanding the subsequent admixture of foreign elements, the remains of the aboriginal inhabitants are still sufficiently distinct to enable their affinities to be traced by their language. Of these, the *Berber* races (from which the north of Africa appears to have received the designation of Barbary) may be regarded as the types; the Kabyles of Algiers and Tunis (the people of Abd-el Kader), the Tuaryks of Sahara, and the Shelahs or mountaineers of southern Morocco, being branches of the same stock. Their dialects vary considerably; but not so far as to constitute distinct languages. The small remains preserved to us of the language of the Guanches, or ancient population of the Canary Isles, appear sufficient to show that they formed another branch of the same great Lybian stock. Now these races are all distinguishable from the Negroes on whose region they border, not only by their language, but also in general by the cast of their features, which is decidedly (to use the common designation) Caucasian; and yet in the southern portion of the country they inhabit, which forms part of the torrid zone, and is not of great elevation, their complexion is often perfectly black, and the cast of features approaches that of the Negro. There is perhaps no better example to be anywhere met with, of the influence of climate in modifying physical characters, than that which is here presented to us within a very limited range of country, and amongst races connected by the closest affinities of language. The inhabitants of the lofty table-land called

Mount Aurasius, although they speak the Kabyle language, are fair and ruddy, and their hair of a deep yellow; whilst the Kabyles in general are of a swarthy colour with dark hair. The tribe of Mozabi, also, has been ascertained by Mr. Hodgson to be remarkably white, though belonging to the Kabyle race. On the other hand, some of the Tuaryk inhabitants of particular Oases in the Great Desert, who are almost as insulated from communication with other races as the inhabitants of remote islands in a wide ocean, have hair and features which approach those of the Negroes; although they speak the Berber language with such purity as to forbid the idea of the introduction of these characters by an admixture of races. That these diversities were not original, but have been acquired under the prolonged influence of external conditions, appears obvious from two considerations. In the first place, the investigations of Professor Newman leave no doubt that the Berber language is to be regarded as a primitive branch of the Syro-Arabian or Semitic group,—a coëval dialect with the ancient Syrian, the Phœnician, &c.; and consequently that we must believe the Berber and other allied tribes to have originally formed part of that stock, and to have migrated westwards, along the northern coast of Africa, long before the first colonization of that coast by the Phœnicians. Secondly, the proper *Arab* tribes, who have spread themselves over the same country, at a date long subsequent to the settlement of the Berber races, and who unquestionably maintain their purity of descent, exhibit very obvious indications of the same influences, though in a less striking degree—as might be anticipated from the comparative shortness of the time during which they have been subjected to them.

To those who maintain the complete isolation of the Negro stock from every other, the characters of the ancient Egyptians and of the nations still inhabiting the eastern portion of North Africa must present peculiar difficulties. "There is no ancient people," says Dr. Prichard, "of whose personal characters, manners, and habits of life, we have half so many testimonies as of those of the Egyptians; and yet there is certainly none respecting whose physical history so much difference of opinion has prevailed." Our sources of knowledge respecting this are at least fourfold; namely,—the testimony of ancient travellers,—the delineations and sculptures executed by the Egyptians themselves,—the mummies preserved in their sepulchres,—and the physical characters of the existing Copts. From the first of these sources it may safely be inferred that the Egyptians were a dark-coloured people, and at the same time that great varieties existed among them, which is the case among the Abyssinians and Hindoos in the present day. The prevailing complexion of the Egyptian nation, as represented by their own artists in those wonderful paintings which have been preserved through thousands of years with all the freshness of the original, would seem to be of a red copper or light chocolate colour; closely resembling that which is at present exhibited by the reddest of the Fulah and Kafir tribes now existing in Africa. The same paintings display their very peculiar physiognomy, which is also transmitted to us in their sculptures; and the impression produced on the mind of an unprejudiced observer by these representations (an impression which may be readily verified by a visit to the magnificent collection which our own British Museum contains) is thus stated by Dénon:—

"Instead of the sharp features, the keen, animated, and restless visages, and the lean and active figures of the Arabian, there were to be seen in the land of the Pharoahs full but delicate and voluptuous forms; countenances sedate and placid; round and soft features; with eyes long, almond-shaped, half shut, and languishing, turned up at the outer angles, as if habitually fatigued by the light and heat of the sun; cheeks round; thick lips, full and prominent; mouths large, but cheerful and smiling; complexions dark, ruddy, and coppery; and the whole aspect displaying (as one of the most graphic delineators among modern travellers has observed) the *genuine African character,* of which the Negro is the exaggerated and extreme representation." (Natural History of Man, p. 152.)

The conformation of the cranium of the ancient Egyptians has been a fertile subject of discussion; from the fact, which the researches of Blumenbach and the more recent inquiries of Dr. Morton of Philadelphia appear to have satisfactorily determined, that a very considerable amount of diversity existed amongst individuals or families of this race. Dr. Morton, like Blumenbach, has been struck with the remarkable differences which present themselves among the Egyptian crania; and he refers them to the Pelasgic or most perfect Caucasian form, to the Semitic type as seen in the Hebrew communities, to the proper Egyptian form, and to the true Negro shape. Besides these typical forms, there are others which present intermediate or mingled characters, as the Pelasgic blended with the Egyptian, the Caucasian mixed with the Negro. About eight tenths of the whole number of crania in Dr. Morton's collection belong to the three forms which he terms Pelasgic, Semitic, and Egyptian; and among these the Egyptian greatly predominates, being as eight to one of the Semitic, and as three to two of the Pelasgic. The Negroid conformation preponderates in about eight instances, or one thirteenth of the whole; and it is to be observed with respect to them, that although the osteological development is more or less that of the Negro, the hair is not crisped but long; so that these crania could not have belonged to genuine Negroes.

There is no reason whatever to believe that the large proportion of Egyptian crania exhibiting the most perfect Caucasian form is to be accounted for by any intermixture of races; on the contrary, there seems to be sufficient evidence for the conviction that one collection of crania from the Memphite Necropolis, in which the proportion of Pelasgic crania was sixteen to only seven of the proper Egyptian form, was of an antiquity long anterior to that of the introduction of the Grecian form under the Ptolemaic dynasty.

The characters of that which is regarded by Dr. Morton as the proper Egyptian type of cranial conformation, from its dissimilarity with either the Negro or the Pelasgic, correspond sufficiently well with the prevailing physiognomy of the national paintings and sculptures; and similar features are still preserved in the Coptic race, which is the nearest representative of the ancient Egyptians, although their descent has perhaps not been altogether pure. In this people many points of decided approximation to the physical characters of the Negroes have been observed by those who have had opportunities of studying them; and amongst these we may cite the testimony of Ledyard, a traveller of the highest intelligence and veracity, without any theory to support, who suspected the Copts to be the origin of the Negro race.

We have thus a coincidence between all the sources of evidence with regard to the physical characters of the ancient Egyptians, so remarkable as to leave but little ground for doubting the conclusion to which they lead, namely, that this nation was more closely united to the Negro race than to any other of the great subdivisions of the human family. Consequently, unless we regard them as having originated from a parentage altogether distinct from that of any of the nations by which they were surrounded, we must believe that they constituted a branch of the great African stock; and thus that they are either elevated forms of the Negro type, or that the Negro races are degraded forms of the Egyptian type. The latter will perhaps appear the most probable hypothesis, when it is borne in mind that, in spite of what appears to be a fundamental dissimilarity of language, the ancient Egyptians and the early inhabitants of India were connected by such numerous and striking features of resemblance in manners and customs, in social and political institutions, in religious observances, and in philosophical belief, as to forbid the idea that such a correspondence can have arisen out of any similarity in external conditions, or can have had its origin in anything else than the same source of mental culture. A remarkable resemblance has also been noticed between the Egyptian features, and those expressed in the most ancient Hindoo sculpture. On the other hand there are many indications of similar relationship between the Egyptians and other African nations; and these relations seem to present themselves also in the general laws governing the structure of their languages. Great difficulties attend the analysis of the ancient Egyptian language, arising from the very imperfect knowledge of it which has been yet attained. The Coptic of the present time is undoubtedly its nearest existing representative; but this has been so changed from its original form by the influence of the various colonizations and conquests which the country has undergone, as to be anything but a trustworthy guide. This much, however, may be stated: that in the tendency to effect modifications in the roots (such as the distinctions of gender, number, and case in nouns, and the various inflections of verbs) by *prefixes,* instead of by terminations as in the great family of Indo-European languages,—a feature of grammatical structure, which (as the readers of our former article will remember) must be regarded as of much greater philological importance than any mere coincidence of vocabulary,—there is a most remarkable correspondence between the Coptic on the one hand and the Kafir (one of the best known of the proper African languages) on the other.

The various nations at present inhabiting the portion of Africa adjacent to the Red Sea exhibit characters which approximate on the one hand to the Negro type, whilst in some respects differing remarkably from it, and on the other side appear to preserve the impress of the stock from which the Egyptians were derived. In fact it is not difficult to show an uninterrupted gradation from the Egyptian to the Negro type, in the several tribes which people the north-eastern portion of the African continent. This gradual change has been supposed by those who regarded the ancient Egyptians as of Caucasian origin, to result from an intermixture of races on the confines of regions originally allotted to each separately. But such an idea is not at all borne out by the close, accurate, and extended observations which have been in progress since the period of the French expedition into Egypt, and which have been of late prosecuted with the greatest zeal by M. d'Abbadie. These intermediate tribes are not Mulattoes, nor do they at all resemble mixed races; but they have each their distinguishing physical features and peculiarities of language, which mark them out as races distinct from the Negroes on the one hand and from the white races on the other, whilst they present points of resemblance to both. Here as elsewhere we find that the lightest complexions and the most elevated physical conformation are to be found among the inhabitants of the highlands; whilst the dwellers on the low plains beneath the same latitudes present a much nearer approach to the Negroes, not merely in the blackness of their skins, but in the thickness of their lips, the flatness of their noses, and the crispness of their hair.

It must be regarded, then, as an established fact that the Negro races are not separated from other branches of the human family by that definite line which has been commonly supposed to mark them out; and we shall presently find that amongst those nations which are usually included in that designation, the varieties of conformation are nearly as great as among the inhabitants of the northern and north-eastern portions of the continent. But before proceeding with this inquiry, the sense in which the word Negro is to be understood must be properly defined.

"It ought to be remembered that the word Negro is not a national appellation, but denotes the ideal type constituted by the assemblage of certain physical characters, which is exemplified in the natives of Guinea in Western Africa, and in their descendants in the West Indies. When these characteristics are not at all found, it has often been said that the African nations, though black, or nearly black, and woolly-haired, are not Negroes. Thus the Kafirs and Hottentots are said not to be Negroes. On the same principle we ought to except the nations of the interior of Africa or of Soudan, in some of which we could scarcely recognise any considerable resemblance in features to the Negroes of Guinea." (Natural History of Man, p. 292.)

There is an extensive region in Central Africa, bounded on the north by the Great Desert, and on the south by a chain of mountains that stretches across the continent about ten degrees north of the equator, which is inhabited by nations comparatively elevated in the scale of civilization. Our knowledge of these, which previously rested on the exaggerated and therefore incredible accounts of the earlier travellers, and on such information as might be transmitted through the border countries with which they trade, has of late been rendered much more precise by the labours of Mungo Park, Denham, Clapperton, Lander, and other adventurous explorers. As might be anticipated, the physical characters of these nations are far more elevated than those of the tribes that have not emerged from the state of degradation in which they have existed from the time of our first acquaintance with them.

"The tribes in whose prevalent conformation the Negro type is discernible in an exaggerated degree, are uniformly in the lowest stage of human society; they are either ferocious savages, or stupid, sensual, and indolent—such are the Papels, Bulloms, and other rude hordes on the coast of Western Guinea, and in the Bight of Benin, countries where the slave trade has been carried on to the greatest extent and has exercised its usually baneful influence. On the other hand, wherever we hear of a Negro state, the inhabitants of which have attained any considerable degree of improvement in their social condition, we constantly find that their physical characters deviate considerably from the strongly marked or exaggerated type of the Negro. The Ashanti, the Súlima, the Dahomans, are exemplifications of this remark. The Negroes of Guber and Haúsa, where a considerable degree of civilization has long existed, are perhaps the finest race of genuine Negroes on the whole continent, unless the Iolofs are to be excepted. The Iolofs have been a comparatively civilized people from the era of their first discovery by the Portuguese." (Physical History of Mankind, vol. ii, p. 338.)

So far as our information extends, it seems pretty certain that here, as in other cases, the relative lightness or darkness of complexion among the different tribes depends in great degree upon the elevation of the country which they inhabit. That external conditions have an effect in degrading or elevating the physical conformation, seems evident from the circumstance that those African races which have the Negro character in an exaggerated degree, and which may be said to approach deformity in person—the ugliest blacks with depressed foreheads, flat noses, crooked legs—are in almost every instance either inhabitants of low countries, often of swampy tracts near the sea-coast, where many of them, as the Papels, have scarcely any other means of subsistence than shell-fish and the accidental gifts of the sea ; or they occupy thick forests in the hollows beneath lofty chains of mountains, whose alpine heights are inhabited by tribes of lighter complexion and more elevated physical characters. This coincidence is so remarkable, that we feel confident that no unprejudiced person who shall attentively trace it out to its full extent, can continue to feel any hesitation as to the fact. The evidence is further strengthened by the changes which may be proved to have taken place in the physical characters of particular races, in consequence of an alteration in their external conditions. Thus the Barabra or Nubians are descended from the Negro mountaineers of Kordofan, a branch of whom settled on the banks of the Nile about fifteen centuries ago, where they have become partially civilized. Their complexion has become much fairer, and their features bear more of the European stamp ; their hair, too, is long and slightly crisp without being woolly. Their descent is attested not merely by historical evidence, but by the community of language which is still traceable ; and there appears to be sufficient reason for the belief that this alteration in their characters is not the result of any intermixture with the Arabs or other inhabitants of the Nile-Valley. A similar change has taken place, apparently under nearly corresponding circumstances, in the characters of the Funge, the conquerors of Sennaar ; who, though descended from the Shilúkh Negroes, have no longer the genuine characters of the Negro race. One of the peculiarities of this last-mentioned nation is the frequent appearance among them of a red complexion and of red hair. Indeed the individuals thus distinguished are stated to form a separate caste ; being known under the name of "El Akmar" or "the Red People." In other parts of Africa the xanthous variety often appears, but does not multiply ; individuals thus characterized being like seeds which perish in an uncongenial soil.

Having thus indicated the chief characters which present themselves in the several nations inhabiting the portion of Africa north of the equator, we must briefly notice those of the southern tribes. Of these, however, with few exceptions, our knowledge is much more limited ; the interior of South Africa having been as yet unexplored by civilized man ; and the only people well known to us being those of a few points on the coast, such as Kongo on the one side and Mozambique on the other, and those of the southernmost extremity, or the region of the Cape. As we pass southwards from the equatorial region, we find a gradual departure from the proper Negro type; and this is the greater according to the degree of civilization, and the general improvement in external conditions. Thus on the sea-coast, and among the more savage races, as the Makua of Mozambique, much of the true Negro physiognomy prevails; but even here a milder and more intellectual expression is observable than among the natives of Guinea. Their hair is woolly, and their colour black; but their skulls are more vaulted and capacious in the anterior part, and have much less of the prognathous character. Many of the people of Kongo, Benguela, and Loango recede even more from the Negro physiognomy than do the inhabitants of the eastern coast. When we arrive at the Kafirs, the race of warlike nomadic people, which inhabits the eastern parts of South Africa to the northward of the Hottentots, we find so great a departure from the Negro type, that many travellers have regarded them as having had a different origin. The degree of this departure, however, varies greatly in the several tribes; for whilst some of them are black and woolly-headed and display the general characters of the Negro (although not so strongly marked as in the natives of Guinea), others recede considerably both in complexion, features, and form of head from the typical prognathous races, presenting a light brown colour, high forehead, prominent nose, and tall robust stature. The thick lips and curly hair are generally retained, however; but the hair is sometimes of a reddish colour, and the features almost European. Even among the tribes which depart most widely from the Negro type, individuals are found who present a return to it; and it is interesting to remark that the people of Delagoa Bay, though of the Kafir race as their language indicates, being degraded by subjugation, approach the people of Guinea in their physical characters. Generally speaking, the Kafirs are a people very superior to the destitute savages who occupy the insulated hamlets of central Negroland. They are associated together in large communities under chiefs or kings; they have considerable herds of cattle, practise agriculture and gardening; and though some are semi-nomadic, living in large camps or moveable towns, others inhabit fixed residences, build good houses, and lay up stores. They are even acquainted with the use of iron and copper, and have the art of working these metals, and of manufacturing articles of use and ornament. In the country of the Tammahas, Mr. Campbell saw fields of corn several hundred acres in extent, near the town of Mashow, which contains 10,000 people. And the same traveller describes the Murútsi as cultivating sugar and tobacco, manufacturing razors and knives of iron almost approaching steel, building their houses with masonry, and ornamenting them with pillars and mouldings.

Here we see a vast advance in civilization, and a great elevation in physical characters, among a people who cannot be considered in any other light than as improved Negroes. To say that they are of a different stock from the true Negroes, is to make an assertion for which there is no ground whatever, and which is disproved by evidence of the most satisfactory kind. For between the most elevated Kafir and the most degraded Negro, every possible gradation is presented to us as we pass northwards and westwards from Kafir-land towards the Guinea coast; so that it is impossible to draw any line of demarcation, or establish a distinction between the two races on their respective physical characters. And all the evidence yet collected in regard to their languages tends to show their fundamental analogy. On this head we shall have more to say immediately.

The following is Dr. Prichard's summary of the facts which he has collected with regard to the physical characters of the proper African nations.

"The dark-coloured nations of Africa do not appear to form a distinct race, or a distinct kind of people, separated from all other families of men by a broad line, and uniform amongst themselves, such as we ideally represent under the term Negro. There is, perhaps, not one tribe in which all the characters ascribed to the Negro are found in the highest degree; and in general they are distributed to different races in all manners of ways, and combined in each instance with more or fewer of the characters belonging to the European or the Asiatic. The distinguishing peculiarities of the African nations may be summed up into four heads; viz. the characters of complexion, of hair, of features, and of figure. We have to remark,

"1. That some races, with woolly hair and complexions of a deep black colour, have fine forms, regular and beautiful features, and are, in their figure and countenances, scarcely different from Europeans. Such are the Iolofs near the Senegal, and the race of Guber or of Haúsa in the interior of Súdan. Some tribes of the South African race, as the darkest of the Kafirs, are nearly of this description, as well as some families or tribes in the empire of Kongo; while others have more of the Negro character in their countenances and form.

"2. Other tribes have the form and features similar to those above described: their complexion is black, or a deep olive or copper-colour approaching to black, while their hair, though often crisp and frizzled, is not the least woolly. Such are the Beshari and the Danákil and Hazorta, and the darkest of the Abyssinians.

"3. Other instances have been mentioned, in which the complexion is black, and the features have the Negro type, while the nature of the hair deviates considerably, and is even said to be rather long and in flowing ringlets. Some of the tribes of the Zambesi are of this class.

"4. Among nations whose colour deviates towards a lighter hue, we find some who have woolly hair, with a figure and features approaching to the European. Such are the Bechuana Kafirs, of a light brown complexion. The tawny Hottentots, though not approaching to the European, differ from the Negro. Again, some of the tribes on the Gold Coast and the Slave Coast, and the Ibos in the Bight of Benin, are of a lighter complexion than many other Negroes, while their features are strongly marked with the peculiarities of that race.

"These observations can hardly be reconciled with the hypothesis that the Negroes are one distinct species. We might more easily adopt the notion that there are among them a number of separate species, each distinguished by some peculiarity which another wants; but on that supposition the deviation will be so gradual from the physical characters of other human races, as to undermine the ground on which the opinion of a specific and strongly-marked distinction has been founded. Separate species of organized beings do not pass into each other by insensible degrees." (Physical History of Mankind, vol. ii, p. 342.)

Our acquaintance with the African languages is extremely imperfect; and, strange to say, the materials are most deficient where we might have supposed that they would be most abundant; namely, in reference to the true Negro languages of the Slave Coast. Putting aside the languages of the nations bordering on the Nile, it is of those of the Kafirs and of the empire of Kongo that most is known. These agree in the most essential characters of affinity; those, namely, which are derived from their grammatical structure. And with these dialects, spoken in the remotest portions of the African continent, the ancient Egyptian seems to be connected by similar relations. Every probability seems to exist, therefore, in favour of a conformity of this nature amongst the African languages in general; although, from the absence of any of those circumstances which tend to *fix* a language or dialect, there may be little correspondence in their vocabularies. If this be the case, it must be admitted to afford a strong corroboration of the opinion that the proper African nations (from which those that are distinctly traceable to the Syro-Arabian stock must be excluded) have had a common origin; and that the ancient Egyptians formed a branch of the same trunk. This view derives further confirmation from the remarkable coincidence in religious notions and observances which may be traced between the ancient Egyptians and the existing inhabitants of remote portions of Africa. Among these may be mentioned the traces of animal worship, the belief in metempsychosis, the observance of circumcision and a variety of other rites, which are described by travellers as existing among the Kafirs, the native people of Madagascar, and some of the tribes inhabiting the western parts of Africa. These, as Dr. Prichard justly remarks, are too extensively diffused, and occur in too many instances, to be attributable to accidental coincidence.

The arguments which are scattered through this most interesting portion of Dr. Prichard's larger work may be thus briefly summed up. The proper African nations must be regarded as having had a common origin, and therefore as belonging to one and the same great family; or they must have had several distinct pairs of ancestors. The latter supposition appears to be entirely refuted by the very gradual nature of the transition between the physical characters of the several nations, which renders it impossible to draw any line between them; and corroborative evidence to the same effect is derived from relations in the languages of remote nations, and from community in religious and other observances. These arguments tell equally, of course, in favour of their community of origin, one with another; and there is no difficulty in accounting on this supposition for the diversities in physical characters which present themselves amongst them, since these diversities are distinctly traceable in many instances to the influence of external conditions, operating through long periods of time, and never transgress the ordinary limits of variation in those species of animals which have a constitution of such a nature as to adapt themselves to different external circumstances. Now if it be once admitted that the proper African nations have a common origin, it is impossible to give assent to the proposition that the original stock is one of decided inferiority to that of the white races; since we find at the present time the closest approximation to the latter, among the more elevated tribes of Africans, both in physical characters, and in moral and intellectual nature; whilst history informs us of the important share which the ancient Egyptians had in the civilization of the world.

In our account of the African nations we have hitherto left out of view the Hottentots and Bushmen, which depart from the general type more widely than the rest; the differences between these and the true Negroes, both in language and physical characters, being such as to lead many ethnologists to rank them as of distinct origin. Of the real identity of the two nations just named, we gave the chief proofs in our former article; and we shall now, therefore, treat them as constituting a single race. Their physical characters present a remarkable mixture of the Negro with that of the Mongolian type. They are thus described by Dr. Prichard, chiefly on the authority of Mr. Barrow.

"'The Hottentots,' says Mr. Barrow, 'are well proportioned, erect, of delicate and effeminate make; not muscular; their joints and extremities small; their face generally ugly, but different in different families, some having the nose remarkably flat, others considerably raised. Their eyes are of a deep chesnut colour, long and narrow, distant from each other; the inner angle being rounded, as in the Chinese, to whom the Hottentot bears a striking resemblance. The cheek-bones are high and prominent, and with the narrow pointed chin form nearly a triangle.'" (Physical History, vol. i, p. 273.)

"The complexion of the Hottentots is like that of the palest Negro, but still more dilute. Dr. Sparrman compared it to the colour of a person affected with jaundice. Mr. Barrow says it is of a yellowish brown, or of the hue of a faded leaf. According to Mr. Burchell, the complexion of the whole race of Hottentots is of a tawny buff or fawn-colour; such as a painter might imagine that of a Guinea Negro would be, if it were half washed off, and a light tint of ochre put over the remainder.

"The hair of the Hottentots is said by Sparrman to be more woolly than that of the Negroes. It is thus described by Mr. Barrow. 'The hair is of a very singular nature; it does not cover the whole surface of the scalp, but grows in small tufts at certain distances from each other, and when clipped short has the appearance and feel of a hard shoe-brush, except that it is curled and twisted into small round lumps about the size of a marrow-fat pea. When suffered to grow, it hangs on the neck in hard twisted tassels like fringe.'" (Op. cit. vol. ii, p. 278.)

There are few skulls belonging to this race in European collections; but such knowledge as we possess in regard to their conformation accords with the foregoing description in the indication of a decided approximation to the Mongolian type. The width of the orbits, their distance from each other, and the large size of the occipital foramen, are points in which there is a remarkable conformity. The sparse distribution of the hair, the large admixture of olive with black in the complexion, and the great obliquity in the apertures of the eyelids, are characters which seem to separate this remarkable race from all the other African nations; and this separation appears further justifiable on the ground of the peculiarities of their language, of which, however, little is yet known philologically. The buttock-hump or steatopyga is a peculiarity supposed to be characteristic of the Hottentot race, which has attracted much attention. It is a well-ascertained fact, however, that this accumulation of fat is by no means universal amongst the Hottentots and Bushmen,—being, as Mr. Burchell remarks, about as frequent as corpulency among European nations; and also that it occurs in other tribes of Southern Africa, as the Makuani of the Mozambique coast. It cannot, therefore, be regarded as affording the least indication of difference of race; but would seem to result from the operation of the same local influences, whatever be their nature, as those which produce a similar accumulation of fat in the rumps and tails of the sheep inhabiting the same regions.

The relation of the Hottentot race to the other African nations is a question of the highest interest; and one which leads us into the most speculative department of ethnology,—that which relates to the earliest dispersion of the human race over the earth. Two views may be taken of their origin. First, we may regard them as branches of the general African stock; and must then look for the explanation of their physical peculiarities in the same kind of coincidence between the operation of external agencies and the inherent tendency to variation, as that which has produced the changes in the conformation of other members of the same family of nations to which we have already referred. Or, secondly, we may regard them as legitimate descendants of the Mongolian stock, which, under the direction of the migratory propensity of that race, might have wandered into Africa at a period anterior to the settlement of the ancestors of the proper African nations; and which were afterwards pressed upon by the latter, until they were at last driven down into the remotest corner of the continent, just as the ancient Britons have been pent up in Wales and Cornwall. The former of these suppositions is evidently that to which Dr. Prichard is most inclined; and it certainly derives a remarkable degree of support from the coincidence between the physical characters of the portion of Southern Africa inhabited by the Hottentot races, and that part of the continent of Asia which seems the proper dwelling-place of the Mongolian stock.

The *karroos*, or hard dry elevated plains of Southern Africa, bear a very striking resemblance to the *steppes* of Central Asia; except that the latter have the advantage of a more abundant and equable supply of water, which of course modifies their vegetation. To the hypothesis that this similarity of conditions may have been the chief cause of the points of resemblance which the Hottentot race bears to the Mongolian type, and that they have really the same descent with the proper African nations, it may be objected that there is not that *gradation* of resemblance which we see between the other most distinct forms of the latter; and that the Kafirs, some tribes of which reside in districts of a very similar nature to the Hottentot country, do not show much resemblance to them in physical characters. That the tendency to variation in the original Ethiopian stock does not run in the direction of the Mongolian type, but leads on the one hand towards the more elevated form of the European head, and on the other towards the degraded type of the true Negro, would appear from the observations of Dr. Morton already cited upon Egyptian crania. The entire difference of language between the Hottentots and Kafirs (so far, at least, as our knowledge of them enables us to judge) would seem another cogent argument against the idea of their family relationship.

In favour of the latter of the two suppositions above offered, may be urged the resemblance in physical characters between the Hottentots and the Mongolian nations, which is so strong as to have led Dr. Knox (who has had ample opportunities of seeing these people in their native country) to regard them as of Kalmuck descent; the chief points of difference being the darker complexion and thicker lips, two characters which we know to be among the first to undergo changes in accordance with external influences. It may be further remarked that there is strong ground for the belief (quite independently of theory) that the Hottentots made their way from the north into their present region along the western side of Africa; and that they arrived at the southern extremity many ages before the Kafirs advanced towards the same quarter by the eastern coast.

However this may have been, the latter people have certainly encroached extensively upon the Hottentots; as appears from the circumstance that the names of rivers and of places now within the territories inhabited by the Kosas and Bechuana Kafirs are plainly derived from the Hottentot language. And this gradual encroachment, by which the unfortunate Hottentots have been dispossessed of their rightful territory and have been degraded, one tribe after another, into the wretched condition of savage Bushmen, has continued ever since this part of the world has been known to Europeans; not only on the part of the lawless but enterprising Kafirs, with whom "might makes right," but also by civilized and christianized Europeans, who profess moral and religious principles that cover their practice in this respect with the deepest shame.

We shall not here enter upon the general argument from analogy in favour of the supposition in question; since it will be more appropriately explained and developed, when we have pointed out the peculiarities in the distribution of nations of undoubted Mongolian descent. But we must stop to remark that whatever view we may take of the origin of the Hottentot race, there is no adequate ground for regarding them as descended from a stock primarily distinct from that which has given origin to other branches of the human family. We have seen that there is nothing in their physical characters which really departs more widely from the general type of the African nations, than these depart from each other; the Hottentots being ordinarily classed with the Negro races by those who have not attended to their peculiar distinctive features. And Dr. Prichard has brought together a most interesting body of evidence, derived chiefly from the accounts of the various missionaries who have devoted themselves to the civilization and christianization of the South African races (the most successful of whom have undoubtedly been the United Brethren), in proof of the fact that there is no essential psychological difference between the Hottentot races, even when degraded to the condition of Bushmen, and those which we are accustomed to regard as far more elevated in the mental scale. We must attempt to estimate the character of the Hottentot race, not from their present degraded condition, after the cruelty and oppression which they have endured from European colonists during so many generations have broken their spirit and reduced them to bondage or exile, but from the much more favorable accounts left by the older writers of the condition of these tribes soon after the first settlement of the Dutch colony, which are singularly at variance with the descriptions given by later writers. Copious extracts from these will be found in both of Dr. Prichard's works; as well as a most interesting collection of evidence as to their capability of receiving religious instruction.

The third volume of Dr. Prichard's Physical History is occupied with his account of the nations of Europe; and the fourth with that of the Asiatic nations. As there is a considerable mixture of races, however, in these two quarters of the world, and as our object is not so much to trace out the history of individual tribes, as to take a general survey of the whole, it will be more convenient for us to group the several nations, not according to the quarter of the globe they inhabit, but according to their agreement in physical and glottological characters. Only two principal types of physical conformation can be discovered amongst the people of

Europe and Asia; those, namely, which are commonly termed the *Caucasian* and the *Mongolian.* Both these names, however, are in the highest degree inappropriate, as will hereafter appear; and we only employ them, in preference to the *Iranian* and *Turanian* of Dr. Prichard, in order that our meaning may be the more readily apprehended by our readers. For the first we may, however, use the term Indo-Atlantic; which expresses sufficiently well the present geographical distribution of those nations which possess, with few exceptions, that type of bodily configuration of which the Greeks seem to display the most perfect model. These nations inhabit the south-western portion of the Asiatic continent,—that, namely, which includes the whole of Hindoostan and the Deccan, as well as Persia and Arabia,—together with the north of Africa, and nearly the whole of Europe. The Mongolian nations, on the other hand, inhabit the centre, northern, and eastern portions of the Asiatic continent, and extend themselves into Northern Europe. We shall afterwards find that the distribution of this race is probably still more extensive.

The skulls of the *Indo-Atlantic* nations generally differ from those of other races in having all their parts moderately developed; and in their oval figure, and the absence of any considerable antero-posterior or lateral projection, they hold a middle place in many respects between the crania of the Negro races and those of the Northern Asiatics. But within the limits of this general form there is a very large amount of variation, both among particular tribes, and among individuals of the same tribe; and many of these variations lead us towards the other types of conformation. In some cases there is distinct evidence that the original type has degenerated. No uniformity whatever exists amongst the Indo-European nations in regard to *colour;* for they present every intermediate gradation between the white or florid hue of the northern Europeans to the jet black of many tribes in Libya and to the south of Mount Atlas. As remarked in our former article, the close relation which we everywhere find in these nations between colour and climate (whether the latter depends upon latitude or elevation above the sea), coupled with the known changes which have taken place in the hue of tribes or nations of whose migration we have historical evidence, leaves no reasonable doubt that this character is one peculiarly liable to variation, and not in the least to be relied upon as an indication of diversity or unity of race. The *hair* is generally long and flexible; but variations present themselves in this respect also, both amongst the larger groups and between individuals. Thus we meet, every now and then, amongst the fairer nations, with individuals having the short, crisp, black hair of the Negro, who have not a particle of Negro blood in their veins; or the dark, straight, but scanty hair of the Kalmuck; whilst, on the other hand, red or auburn hair is not uncommon in individuals or even whole families amongst nations in which the characteristic hue of the hair is black.

This great subdivision of the human family must be further separated into two groups of nations; which, though they agree with each other in their general physical conformation, differ entirely as regards their language. And this difference existed in the remotest periods of which we have any historical or even traditionary knowledge. The first of these groups consists of the *Syro-Arabian* nations, which anciently extended over the south-western portions of Asia (including the countries lying between the Euxine and the Indian Ocean, and bounded on the east by Armenia and Persia), and early spread themselves over Northern Africa. The bond of union amongst these nations is their language, which differs altogether from that of all other human races, whilst its varieties are much more closely allied to each other than we usually find elsewhere. In the opinion of Baron Larrey, who has had ample opportunities of observation, the skulls of the existing Arabian race furnish at present the most complete type of the human head; combining the fullest development of the cranial cavity, with the most perfect organization of the parts subservient to the senses: and he states that experience has proved to him that their intellectual perfectibility is proportional to this higher development of physical organization, a great aptitude for acquiring knowledge being united with extraordinary acuteness in the use of the organs of sense. We find, however, that the habits and manners of different branches of this family of nations have varied very widely, from the earliest period of which we have any account of them; some being nomadic, others agricultural, and a third class devoted to foreign commerce and domestic manufactures. Their physical characters, also, have undergone considerable modifications. The varieties of language afford the best means of dividing this assemblage into subordinate groups; of which the following are the principal.

"1. The northern and eastern branch, termed *Aramæan* or *Syrian.* The Syriac of the versions and the Chaldee of the late Scriptures of the Old Testament and of the Targums, are specimens of this language from early times. If the Cappadocians were really Syrians, this was doubtless their idiom. It appears to have been the original idiom of the Hebrews, until the Abramidæ occupied the Promised Land in Canaan, and adopted, as it would appear, from its previous inhabitants, the Canaanitish or proper Hebrew.

"2. The Hebrew, Canaanitish or Phœnician, for they were the same or very nearly the same, as Gesenius has lately proved, was spoken by the Hebrews from the time when they adopted it on their arrival in Palestine to the Babylonish captivity, when they are supposed to have exchanged it for Chaldee, or to have returned to the use of a dialect more akin to their primitive ante-Abramic speech. This language, with perhaps some very slight variations, was the idiom of the Sidonian and Tyrian states, and of Carthage and the Carthaginian settlements. Even the language of Numidia is supposed by Gesenius to have been a pure or nearly pure Hebrew.

"3. The third division of Syro-Arabian dialects are those of the Arabic properly so called, including the Mogrebbyn or Western Arabian language.

"4. It is supposed that a fourth language, belonging to the Syro-Arabian stem, has been discovered lately in the southern parts of Arabia. . . . . These discoveries render it probable that an ancient language cognate with the Syriac, and the Hebrew, and Arabic, but distinct from all, and having a character of its own, once prevailed over an extensive region to the southward of that occupied by the proper Arabic." (Natural History of Man, pp. 141-4.)

It has been also rendered almost certain, through the analysis made of the *Berber* language of North Africa by Professor F. Newman, that this is to be considered as a fifth cognate dialect of the great Syro-Arabian stock; although it differs more from the rest than they do from each other, probably on account of its complete separation from them at an earlier period. And it even appears from the acute researches of the same accomplished scholar, that the languages spoken by some of the Negro nations of Central Africa are certainly, though more distantly, related to the Syro-Arabian stock.

The *present* distribution of the Semitic nations (as they are frequently but erroneously termed) does not at all correspond with the above subdivisions, which express their condition at the earliest period of which we have any knowledge of them.

"Of the several nations who are connected by this community of language, some who were formerly celebrated have become nearly extinct; while others have spread themselves, either as the exiled followers of a persecuted faith, or as the conquering apostles of a victorious one, over the world, and seem destined, through the energy of their invincible mind, to survive to the end of time. The Syrian race scarcely exists: their language only survives in some districts on the borders of Kurdistan; everywhere else they have been lost under the predominant Arabs. The Homerites in Arabia, if there they exist, are little known; the Abyssinian Homerites are the only inhabitants of the province of Tigre, to the eastward of the Tacazze, whose idiom still resembles the ancient Gheez. The Arabs, who spread Islam by their victories from the Atlantic to the Ganges, and the Jews, who are wanderers over the whole world, are perhaps now more numerous than were even their forefathers." (Natural History of Man, p. 145.)

We referred in our former article (p. 69) to the important facts in regard to the influence of climate on complexion, resulting from the comparison of the different branches of the Jewish race, now dispersed through so many diverse climates, yet preserving so completely their national distinctness; and when treating of the nations of Syro-Arabian descent in Northern Africa, we have found the same general correspondence to hold good. A detailed survey of the characters of the proper Arab branch, now so extensively diffused, would lead to the same conclusion.

The *Indo-European* group of nations is more widely spread, and consists of more numerous tribes, than the preceding. Nations who speak languages of cognate origin, and who are proved by that connecting bond to be descendants of one original stock, are now spread from the mouth of the Ganges to the British islands and the northern extremities of Scandinavia. This stock seems to have been early divided into two primary branches: the *northern* or Median; and the *southern* or *Indian*. Between the original languages of these races a marked resemblance can be traced; the *Zend*, or earliest idiom of the Medes, Persians, and Bactrians, being intimately related to the *Sanskrit* or ancient language of Hindoostan. And the traditions of both races point to contiguous regions as their original seat; the earliest records of the Persians indicating that they migrated westwards from a spot in the ancient Bactria, not far from Balkh, to the westward of the Indus; whilst the traditions of the Brahmans refer the origin of the Hindoos to the north-western part of the country lying between the Himalaya and the Vindhya mountains, whence they afterwards moved eastwards and southwards into the peninsula of India. Both the Median and Indian nations, moreover, originally designated themselves as *Arians;* a term of which Dr. Prichard suggests the use, as a concise appellation for the Indo-European group of nations.

The physical history of the Hindoo nation, which must be regarded as the nearest representative of the ancient Indian branch, is interesting, as presenting within moderately narrow limits great diversity in those points of conformation, which some have held to be most permanent and definite characters for the classification of the human races into distinct species. Many circumstances favour this diversity; especially the distinction of castes, which perpetuates the same mode of life in particular families from generation to generation; and the marked differences of climate between the several regions, as when we compare the mountainous elevations of Kashmir and Kafiristan with the plains bordering the great rivers of India. In many instances the origin of the varieties which we encounter can be clearly traced by historical evidence, as well as by affinities of language and conformation; and it cannot be questioned that Hindoos as black as Negroes, others of a copper colour, others little darker than the inhabitants of Southern Europe, and others of a fair complexion with blue eyes and auburn or even red hair, have all had a common parentage; some having become darker than their ancestors, and others lighter, generally in accordance with changes in their residence and habits. There would also seem to be a stronger tendency to variation in complexion amongst families or individuals of the same descent and living under the same circumstances, than exists in the fairer nations. It is especially, however, among the high caste people of the northern parts of India that the fairest complexions are to be met with.

Of the ancient Median race, the main or *Persian* stock has fallen under Turkish domination; and the so-called Persians of the principal towns are in reality Turks. But a large part of the country is still inhabited by the descendants of the ancient Persians, who are termed Kuzzilbashes by the Turks. There are several races inhabiting the borders of Persia, but not properly belonging to the Persian nation,—though probably cognate with them,—which present strongly-marked diversities of conformation, analogous to those already noticed. Among these are the Affghans, inhabiting the mountainous region to the northward of the low country of the Punjab or plain of the Indus, speaking a dialect derived from the ancient Zend, and having every variety of complexion from that of the dark Indian to that of the fair European; the Kurds, inhabiting the high mountainous tract, intersected by deep valleys, which lies between the great upland of Persia and the plains of Mesopotamia, divided into many tribes, which differ from each other in language and in degrees of barbarism and improvement, but which have a peculiar idiom which shows them to belong to the Arian race; and the Armenians, now constituting a numerous and civilized people, connected by early tradition with the Medes and Persians, speaking an idiom allied to the most ancient dialects of the human race, and remarkable for their fine forms, fair complexions, and regularity of features.

The collective body of *European* nations are, as it is now almost universally admitted, a colony, or a series of colonies of the Arian or Indo-Median race. The evidence of this position is chiefly derived from the fact that the European languages, in spite of their diversity, constitute but one philological group; being united not merely by their community in some of the most important roots or primary words, but also by the general similarity of their grammatical construction; and that they obviously have either the Sanskrit or the Zend as a common source or fountain. The conclusion, being strengthened by the general similarity in cranial conformation which presents itself, with subordinate differences, amongst all the nations to which we should thus attribute an Arian origin, becomes almost irresistible; although neither history nor tradition enable us to trace with any certainty at what time nor by what path the Arian colonisers passed into Europe. The European branches of the Indo-European stock are arranged by Dr. Prichard, by the combined assistance of historical and philological evidence, under the following heads:—1. The *Celtic* nations of Western Europe, represented at the present time by the Irish, Scots, and Manks, which form one subdivision; and by the Welsh, and Armoricans or Bretons, which constitute another.—2. The *Germanic* family, which, in like manner, consisted of two principal subdivisions; the Northmen, or ancestors of the Icelanders, Norwegians, Swedes, and Danes; and the proper Teutonic, from which sprang the Saxon or Western German, the Suevian or High German, and the Gothic or Eastern clan.—3. The *Pruthenian* or Old Prussian, including the Lettish, Lithuanian, and Pruthenian proper.—4. The *Slavic* or *Slavonian*, consisting of a western and an eastern branch; from the former of which sprang the Poles, Bohemians, and other nations near the Baltic; and from the latter the Russians, Servians, and other tribes.—5. The *Italian* family of nations, including the Umbrian, Oscan or Sabine, Latin, Sicelian or Œnotrian, and other people of Italy, with the exception of the Tuscans.—6. The *Rassenian* or *Tuscan*, who were a people of different physical characters from the other inhabitants of Italia, and spoke a language that seems to have had little or no affinity to any of the other dialects of the Peninsula.—7. The *Illyrian*, from which have descended the Thracians, Arnaouts, and Albanians.—8. The *Hellenic*, including the nations of ancient Greece, which formed the cradle for the development of most of the elements of modern civilization; and in which the intellect first obtained a permanent predominance over mere animal power. The classical languages of Greece and Italy appear more referable to the Sanskrit or Indian stock than to the Median; whilst the Germanic languages seem rather to have originated in the latter. Of all the extant European dialects, the Lettish and Lithuanian approach most nearly to the ancient Sanskrit. These resemblances, however, cannot be held conclusive with regard to the origin of the several European races in one or the other of the great Asiatic branches; for it is a well-established fact, that each member of the Indo-European class of languages bears traits of particular affinity, or rather of peculiar resemblance, to nearly every other member. Each also contains an admixture of a barbaric or foreign element; the proportion of which varies in different cases. This admixture may possibly have been derived from the languages of the people who inhabited Europe at the time of the first immigration of the Arian races; since there is much ground for the belief, as we shall hereafter see, that this quarter of the globe was by no means in a vacant state at that period, although the traces of the previous population have been generally lost.

Putting aside for a time the question of the mutual relationship of the nations which at the present time inhabit the central, northern, and eastern parts of Asia, and of those which we find still holding to certain countries in Northern Europe, or whose traces we meet in the south-west, Dr. Prichard includes them under the general term of Allophylian races.

"The Allophylian races are spread through all the remotest regions of the old continent, to the northward, eastward, and westward of the Iranian nations, whom they seem everywhere to have preceded, so that they appear, in comparison with the Indo-European colonies, in the light of aboriginal or native inhabitants, vanquished and often driven into remote and mountainous tracts by more powerful invading tribes. The latter seem to have been everywhere superior to them in mental endowments. Some of the Indo-European nations, indeed, retained or acquired many characteristics of barbarism and ferocity; but with these they all joined marks of an early intellectual development, particularly a higher culture of language as an instrument of thought as well as of human intercourse.

"Though rude in respect to many of the arts of life, the Indo-European nations appear to have brought with them a much higher degree of mental culture than the Allophylian races possessed before the Iranian tribes were spread among them. Even the most simple of them had a national poetry, and a culture of language and thought altogether surprising, when compared with their external manners and condition, as far as they can be known or properly estimated. They had bards, or scalds, vates, *ἀοιδοὶ,* who, under a divine impulse were supposed to celebrate the history of ancient times and connect them with revelations of the future, and with a refined and metaphysical system of dogmas, of which it is difficult to imagine the original source. Among these, in the west as well as in the east, the metempsychosis held a conspicuous place, implying faith in an after-life of rewards and punishments, and a moral government of the world. With this was connected, among most if not all the Iranian nations, the notion that the material universe had undergone, and was destined to undergo a repetition of catastrophes by fire and water, and to be renewed in fresh beauty, when a golden age was to commence, destined in its turn to inevitable corruption and decay. The emanation of all beings from the soul of the universe, and their refusion into it,—a doctrine which appears to have formed an essential part of this system wherever it was preserved in a tolerably entire state—borders closely on a species of pantheism and fatalism; and is strongly contrasted with the theology of the Syro-Arabian nations, who only of all mankind appear in the early ages to have recognised the existence of an extra-mundane God, and a real maker and originator of the universe. The Iranian system, which was a religion of poetry and philosophy, and which everywhere produced an abundant growth of mythology, was still more strongly contrasted with the superstition of Shamanism, connected with a belief in sorcery and spells, and the rude materialism which prevailed among all the Allophylian tribes. This last form of superstition, resembling in many particulars the fetissism of Africa, appears to have differed in different parts of Europe and Asia, among the rude aborigines of which it was once universally spread. It has given way, in most instances, to the influence of more systematic modes of belief, introduced by more polished nations; but Buddhism, which is a form of the Indo-European system, has not extinguished in China and Japan the original superstitions of Tao-sse and Sin-mu; nor did Islam, though early adopted by the whole Turkish race, triumph over all the native superstitions of Siberia. Among all nations of Asia and Europe, we discover an order of persons who were venerated as mediators between the invisible powers and their fellow-mortals; but the priests, whether Druids, or Brahmans, or Magi of the cultivated nations, were revered as the depositories of ancient sacred lore, of primitive traditions, of the will of the Gods expressed of old to the first men and handed down, either orally in divine poems, or preserved in a sacred literature known only to the initiated; they were the constituted intercessors between weak mortals and the powers which govern the universe, and which they only knew how to approach by ordained rites. In most instances they were an hereditary caste, into which none were admissible who were aliens to the sacred race. Far different were the twice-born sages of the Hindoos, who sprang from the head of Brahma to govern the multitude that issued from his legs and feet, from the sorcerers or shamans of the northern worshippers of fetisses, who by horrible distortions, cries, and yells, by cutting themselves with knives, by whirling and swooning, assumed the appearance of something preternatural and portentous, and impressed the multitude with a notion that they were possessed by demons. Of this latter description were the wizards of the old Finnish races, whose successors, the sorcerers and witches of Lapland, sell wind to the English mariners. Such were the angekoks of the Esquimaux, discovered by the missionaries to Greenland; and such are the shamans of all the eastern and northern countries of Asia, whither neither Buddhism nor Islam have yet penetrated. By such traits as these, which display more fully and certainly than external manners and the modes of sustaining life, the culture or rudeness of the mind, the barbaric tribes, dispersed over all the extreme parts of the ancient continent, are distinguished from the cultivated nations of Upper Asia, and from the European races allied to them in language and descent." (Physical History of Mankind, vol. ii, pp. 9-13.)

We have given this extract in full, because it shows how particular forms of religious belief may become, when we look below their superficial resemblances or differences and investigate their fundamental characters, a most important means of tracing affinity amongst remote nations. Any assistance of this kind is most welcome as an aid to the ethnologist in his investigations into the mutual relationships of the Allophylian nations, whose history is involved in greater obscurity than that of the tribes which belong to the Indo-European family; the sources of information respecting them being more scanty and difficult of access, and in many instances remaining yet unexplored. We shall commence with a brief account of that group of nations which are commonly regarded as forming the *Mongolian* race; being characterized, more or less decidedly, by the pyramidal form of the skull, and by the xanthous or olive complexion. The original seat of these nations appears to have been the great central plain of Asia, in which all the great rivers of that continent have their sources, whether they are afterwards to flow northwards into the Arctic ocean, eastwards to the sea of Okhotsk and Japan, or southward into the Indian ocean. This extensive district, constituting perhaps one fourth part of the whole area of the Asiatic continent, seems to have been tenanted originally by nomadic tribes, closely resembling in their physical characters, their mental habitudes, and their mode of life, those which still inhabit the same region. The increase of population, however, caused various offsets to diverge from this centre; and these have undergone a greater or less degree of modification, according to the length of time which has elapsed since their separation, and the degree of change in the external conditions to which they have been subjected. Some of these migrations effected or gave occasion to some of the most remarkable revolutions recorded in history; and there seems good reason to believe that similar excursions, at earlier periods of the history of the race, were the means of spreading a human population through countries as yet untrodden by the foot of man. In addition to the physical characters which have been already mentioned as distinguishing these nations, we may stop to remark that the greater proportional width of the head at the plane of the zygomatic arches affords room for greater development of the organs of sense; and in correspondence with this, the pastoral tribes of Northern Asia display a remarkable perfection in the sensitive faculties.

"The Kalmucks especially, according to Pallas, have the finest sense of smelling, the most perfect hearing, and a singularly penetrating and extensive sight; they see objects on their steppes with the naked eye which are invisible to Europeans even with the aid of glasses, and they discover, by smell, fires or the scent of a camp at a surprising distance; and by their hearing they recognise at a remote space the movement of an enemy or of a herd of cattle." (Physical History of Mankind, vol. iii, p. 407.)

Now, it is not a little remarkable that the Hottentots are especially characterized by the possession of this same acuteness of the senses, especially that of sight, which has been noticed in almost precisely the same terms by Europeans whose expeditions they have accompanied. Whether we regard this coincidence,—like the resemblance already noticed in the form of the head, the position of the eyes, the tawny complexion, and the scantiness of the hair,—as indicating an identity of race between the Hottentots and Mongolians, or as resulting from the similar influence of corresponding external conditions, the fact is remarkable and instructive. The American Indians, too, are well known to possess remarkable acuteness of the special senses.

The group of nations whose origin from the central Asiatic plateau seems most distinct, appears to have early consisted of five tribes, who spoke languages which were long regarded as altogether dissimilar, but which are now known to have a common foundation. Of these races three—namely, the Turkish, Mongolian, and Tungusian—occupied the central portion of the plateau; the north-western border was peopled by the Ugorian or Ugrian race, which was probably an early offset from the central stock, retaining least affinity with it; whilst the south-eastern was tenanted by the Bhotiya or mountain people, whose descendants border upon Hindoostan.—The *Ugorian* or *Ugrian* race seems to have extended itself in a north-westerly direction at a very early period, since we find its several tribes in possession of the whole region extending from the Baltic to the rivers Obi and Irtish in Siberia, in times long anterior to the arrival of the German and Slavic nations in the north of Europe. The western branch constitutes the peculiar people still tenanting the northern Baltic countries under the names of Fins or Lappes; to the relative degrees of civilization of these two people of similar origin, and the change in physical characters which has taken place in the former, we have already adverted (p. 75). The people called by the Russians *Tschudes* were of the same stock. Further eastwards the name of *Ugrians* predominated; and these gave origin to the designation of Ogres, which were prototypes of savage monsters, dwellers in forests and mountains, whose name is better known in its fabulous than in its historical import. From the eastern branches of this race, namely, the Vogouls of the Uralian mountains, and the Ostiaks on the Obi, are descended the Magyars or Hungarians, a warlike and energetic people, unlike their kindred in the north; in whom a long abode in the centre of Europe has developed the physical and mental characters of the Arian race, proving the (so-called) Mongolian stock to be susceptible of the highest culture.

The *Turkish* tribes have been commonly but erroneously termed Tartars; the real Tartars (or rather Tatars), however, were a people belonging to the proper Mongolian tribe. The relation of the Turks of Europe and Western Asia to the tribes which still preserve their nomade life in the vast regions that lie north of Persia and Hindoostan has been already discussed (p. 74), and we shall therefore pass on to speak of the proper *Mongolian* division. This constitutes but a very small part of the entire group; for though it covers an extensive tract of country, reaching from Lake Baikal to the north-western boundary of China, yet the people are insignificant in numbers when compared with the Turkish tribes. They still everywhere retain their nomade habits, and exhibit the physical characters of the group in a very well-marked degree, and with considerable uniformity. The *Tungusians* wander over the immense mountainous regions which extend north-eastward from Lake Baikal to the sea of Okhotsk, and are also dispersed along the courses of the great rivers which discharge themselves into the Icy Sea. The tribes within the limits of the Chinese dominion bear the general name of Mantschu (or Manchew), and are improperly called Tartars; the remainder form part of the Russian empire. The former established themselves, in the sixteenth century, as the conquerors of the Chinese empire, and have continued to govern it ever since, and although they still retain some of the characters of the nomadic Tungusians, these are usually much softened down, under the influence of a settled and more civilized life. The *Bhotiyahs* are the nation, often termed Tartars, who inhabit a great part of Tibet and the Himalayan chain; they have a strongly-marked Mongolian countenance; but in vigour of body and in stature they are very superior to the nomadic races to which they are related.

It is very probable, though the fact (from the paucity of information hitherto brought to bear upon the subject) cannot be yet regarded as proved, that all the other tribes on the northern border of Asia are allied to the same class of nations.

The vast region of Asia forming the south-eastern corner of that continent is inhabited by races of people who resemble each other so strongly in moral and physical peculiarities, and in the general character of their languages, as to give rise to a suspicion that they all belong to one stock, which may be designated as that of the Indo-Chinese nations. Under this category may be included the *Chinese*, which have long been the most numerous and powerful of the group, the *Koreans*, and the *Japanese*, all of which present the physical characters of the Mongolian type, though in a form more or less mitigated and variable. The races of the great *Indo-Chinese* peninsula constitute another subdivision, intimately related to the Chinese, both in language and physical characters; such are the people of Siam, Cochin China, Kambogia, Pegu, &c. The physical characters of these tribes present much of that departure from one constant original type, which we elsewhere notice in people that are making advances in civilization. The great peninsula of Hindoostan appears to have been peopled long previously to the immigration of the Hindoo or Arian race; and remains of the aborigines are still found in the hilly parts of Northern India, in the Deccan, and in Ceylon. They constitute numerous tribes which are now for the most part isolated from each other, and which seem to have nothing in common; and very different degrees of civilization are to be met with among them. Thus, of the tribes making up the aboriginal population of Southern Ceylon, namely, the Cingalese, the Kandians, and the Vaidahs, the two former are considerably advanced, in proportion to the last which remain in a state of complete barbarism. Yet there is evidence derived from affinities of language, peculiar customs and traditions, &c., that they had a common origin. Another important aboriginal race is the Tamulian, inhabiting the northern part of Ceylon and the southern portion of the Deccan, and having offsets in different parts of India. Numerous tribes not of Hindoo stock are also found inhabiting tracts of no great extent in the valley of the Brahmaputra, and in the countries near the mouth and lower course of that river and the borders of the Bay of Bengal.

The relations of the Indo-Chinese nations to the great nomadic group of Central Asia are by no means clear. The physical characters of the Chinese would decidedly indicate their descent from the Mongolian stock, and their early history refers to the eastern border of the great central plateau as their original seat; but the Mongolian type is, as it were, softened down or improved; and in many instances the countenance and form of head approach those of Europeans. This departure is still more evident in some of the tribes of the Indo-Chinese peninsula, which are still connected with the Chinese nation by affinities of language. And among the aborigines of Hindoostan, some present the Indo-Chinese form, whilst others approximate the Hindoos. It is not difficult to account for these changes, on the supposition that they have been induced in the races most remote from the central stock by the alteration in their external conditions, just as we find similar changes to have been effected in the Turks, Fins, and Magyars. It is curious to observe that, in all instances, the advance seems to be towards the European or (so-called) Caucasian type. Notwithstanding the indications of affinity, however, to the great central Nomadic group, the great dissimilarity in the languages of the Indo-Chinese nations (of which the Chinese may be taken as the most perfect example) to those of most other Asiatic nations, interposes a great difficulty in the way of the determination of their actual relationship. These languages have been sometimes termed, for the sake of distinction, *monosyllabic;* but this term does not express their real peculiarity; since the *roots* of most other languages are generally monosyllabic, words of two or more syllables being usually either compound (that is, made by the union of two or more roots), or formed by inflections of the roots.

"The main peculiarity of the monosyllabic idioms consists, as M. de Humboldt observes, in the double circumstance of the total want of affixes or syllables subjoined for the purposes of inflection, and the peculiar manner of pronunciation, by which, even when the mind connects ideas, the sounds of syllables are left separate, and, by a distinct intonation or accentuation, still retain the character of individual or particular words. It has been remarked by the great philologer whom I have just cited, that the Chinese and the Sanskrit languages exemplify the two most opposite methods of construction. The Sanskrit denotes all the relations and connexions of words and of ideas by grammatical forms, written and expressed in pronunciation; the Chinese leaves the perception of these relations to be the work of the mind. The use of some particles being excepted, of which the Chinese can, however, in a great degree dispense, this language expresses all grammatical relations of words by mere *position,* fixed according to certain invariable rules, and by the explanation of sense which the context or connexion of the sentence supplies." (Physical History of Mankind, vol. iv, p. 541.)

The perpetuation of this kind of language appears to be partly a result of the use of hieroglyphic symbols instead of alphabetic writing; each of these symbols standing for a distinct word, which would thus continue to be remembered and recognised in its individual sense. Now although there is so wide a separation between the Chinese and allied languages, and those of the Nomadic tribes of Central Asia, yet the Bhotiya or ancient Tibetian language seems to present relations to both; so as to warrant the conjecture that the Indo-Chinese nations are really offsets from the great central stock, the Bhotiya probably representing their original form. The similarity of the Tamulian language of Hindoostan to the same type justifies the same conclusion with regard to some at least of the aboriginal tribes of the Indian peninsula. That small groups of aborigines, separated from each other at a very early period, before their language had become fixed by advances in civilization, should gradually lose this connecting link by the almost entire alteration in their vocabulary which would take place in successive generations, under the influence of new circumstances, and more particularly from intercourse with surrounding people speaking languages of an entirely different character, cannot be regarded as unlikely. Whether any traces of fundamental connexion can yet be discerned amongst all the languages of the supposed aboriginal tribes of Hindoostan, we have no sufficient data to determine.

Passing from south-eastern Asia to the extreme western border of Europe, we find on the flanks of the Pyrenean range the remains of a people now known under the name of the Basques or Biscayans. There is no doubt that these are the representatives of the ancient Iberians, a people who inhabited the northern coasts of the Mediterranean, from Italy westwards, before their occupation by the Celtic nations. The national appellation of these people in their own idiom is *Euskaldunes;* and they term their language the Euskara or Euskarian speech. This language has been attentively studied, especially by the late celebrated Baron William von Humboldt; who, during a residence in Spain, devoted himself to this subject, and to the collection of materials illustrative of the ancient literature of the Iberians. He has hence come to the conclusion, which corresponds with that founded upon other data, that the Iberians belong to the very earliest stock of European nations; and so far from their language having been derived (as some writers have supposed) from the Celtic, it must have been in existence at a period long anterior to the migration of the Celtic nations into Western Europe. But the Euskarian has some remarkable traits of resemblance to the Finnish language, and thence to the general family of languages in High Asia; corresponding with them in two remarkable peculiarities,—the first being the absence of difference of gender in nouns substantive, and the second being the practice of subjoining to nouns all particles which modify their meaning, and to verbs and clauses all the pronouns personal and even relative. But it differs from the High Asiatic languages in the variety of its inflections, and in the extensive use of auxiliary verbs; as well as in its tendency to form words by *agglutination,* which has been noticed as so remarkable a peculiarity of the American languages. (p. 53.) It must be borne in mind that, as M. de Ponceau has observed, the Euskarian language, "preserved in a corner of Europe by a few thousand mountaineers, is the sole remaining fragment of perhaps a hundred dialects, constructed on the same plan, which probably existed and were universally spoken at a remote period in that quarter of the world." We should do very wrong if we were to attempt to estimate the former extent of the Iberian nations by that of the existing representatives of them. From the manner in which they are spoken of by the ancient geographers and historians, there is good reason to believe that the Iberi inhabited even in their time the greater part of the peninsula of Spain and the southern parts of Gaul; and this opinion has received the most satisfactory confirmation from the analysis which M. de Humboldt has made of the local names throughout the peninsula, as well as in the countries beyond its limits, which are said to have been tenanted by people of the same stock with the Iberi.

We think it impossible to give a candid consideration to these most remarkable facts, without coming to the conclusion that a large part of Europe was inhabited by aboriginal tribes descended from the nations of High Asia, anteriorly to the north-westerly migration of the tribes of the Indo-Median stock. The irruption of these tribes into Central Europe seems to have separated the aborigines into two great divisions; of which one was gradually pressed northward and eastward, so as to be restricted to Finland and Lapland; and the other southward and westward, so as to be confined at the earliest historic period to a part of the peninsula of Spain and the south of France, gradually to be driven before the successive irruptions of the Celts, Romans, Arabians, and other nations, until their scanty remains found an enduring refuge in the fastnesses of the Pyrenees. The Magyars of Hungary seem to be the only descendants of the High Asiatic stock that have maintained their footing in Central Europe; and this may have been due to their having made greater advances in civilization,—if we may judge, at least, from their present rank in the scale of nations, and the corresponding improvement in their physical characters.

To those who have been accustomed to the use of the term *Caucasian* as designating the most improved type of physical structure, or as indicating the supposed original seat or centre of the races which exhibit that type, it will appear very strange to be informed that the proper Caucasian and Georgian race is made up of a group of nations which has been confined, from the earliest periods at which we have any knowledge of them, to the mountainous regions in the neighbourhood of the great Caucasian chain; that these nations differ so completely in language and manners from all those by which they are surrounded, that it is difficult if not impossible to settle their true descent; and that of the numerous tribes of which they consist, some are even unintelligible to each other. They agree, however, in their warlike habits and in their hatred of foreign domination; which, with the advantages of their position, have enabled them to maintain their independence against Persians, Greeks, Romans, Mongolians, and Turks, and even to keep at bay the armies of Russia. The reputation which they have acquired for personal beauty is probably much beyond the truth; those individuals only being selected for the Turkish slave-market who are distinguished in this respect. We are told by Pallas, that both males and females are better formed than are the people of most other unpolished nations, although the women are by no means uniformly "Circassian beauties;" but other travellers inform us that glaring red hair is by no means unfrequent among them. The languages of the several Caucasian nations, although so unlike one another as to check their mutual intercourse, have yet that species of affinity which evinces a common origin at some very distant era. With the Indo-European family of languages they seem to have no relation whatever; and their correspondence in physical characters cannot, therefore, be admitted as a proof of similar descent, any more than it is in the case of the Turks, Magyars, or Biscayans. The only relations which the

Caucasian languages present to other known forms of speech, are to the dialects of Northern Asia, particularly those of the Finnish nations and of the Samoiedes. With these they correspond alike in the coexistence of numerous roots, and also in the characters already noticed as common to the Euskarian tongue and the languages of High Asia. Hence, upon the general principle of giving more weight to fundamental affinities of language (especially when there is evidence that these cannot be accounted for by intercourse of the nations which present them) than to resemblance in physical characters, we should be led to the remarkable conclusion that the Caucasian races are really descended, like the Eskaldunes, from the Northern Asiatic stock; and there is nothing in their present aspect or past history to negative such a conclusion, although the evidence upon which it rests must be admitted to be very imperfect.

The fifth and last volume of Dr. Prichard's great work includes the Oceanic and American races. The length to which our survey has already extended requires that our notice of these should be comparatively brief.

The name of *Oceanica* has been given by Malte-Brun and other geographers to the great Southern Ocean, with the numerous islands and groups of islands within its limits. Its boundaries are very wide; for it is regarded as extending from the eastern coast of Africa, where Madagascar is the first land it includes, to the western shores of America, the last or most easterly land it embraces being Easter Island. In latitude it extends from the coast of Asia southward without limit, including all the insulated lands that have been discovered in the Austral seas; and it is also considered to embrace the greater part of the Northern Pacific Ocean and the islands scattered through it. The Kurilian and Aleutian Islands are not usually reckoned as belonging to it, because they are known to be inhabited by races of people who came immediately from the adjacent continents, and who are unconnected with those tribes of the human race who peopled the remote islands of the great ocean. The most northerly groups within the circuit of Oceanica, and inhabited by its peculiar races, are the Sandwich Islands in the eastern part, and the Marian Islands or Ladrones, and the great Archipelago of the Carolines to the west. In this great region, which presents such immense varieties of climate and soil and geographical position, we should expect to find, if anywhere, great diversities in the physical characters of the human tribes to which it affords habitation. The following is Dr. Prichard's general account of them:

"I propose to distinguish the whole collective body of these native races of the Great Ocean by the name of Oceanic or Pelagian nations. Polynesian they have often been called, but that term has been applied of late to a particular division of them, and is no longer fit to be used as a general name. Under this appellation of Oceanic races several different descriptions of people are comprehended.

"The whole collective body of these native races of the Great Ocean are termed Oceanic or Pelagian nations. They are divided into three principal groups. The first, which alone can be described with propriety as a particular race or family of nations, comprehends the numerous and widely-dispersed Malayo-Polynesian tribes, who, though in some instances displaying certain diversities in physical characters and manners, are proved by a decided affinity of dialects to be originally of one kindred. Next to these we must place a group of nations who are very inferior to the Malayo-Polynesians in arts and civilization, and differ from them remarkably in physical characters. These nations also differ from each other in stature and bodily conformation; the characteristic which is common to them is a certain approach in colour, features, and particularly in the nature of their hair to the Negro races of Africa. In this last particular there is, however, a great difference among them. Some tribes, as the Papuas of New Guinea, have spiral and twisted hair growing in large tufts to a considerable length, which, when combed out, forms an immense frizzled mass enveloping the head with a sort of periwig of great circumference. Other tribes have hair growing in short and closely frizzled curls like the Negroes of Guinea. We cannot comprise all these nations under the designation of Papuas, which belongs to a particular division of them. I shall term them, for the sake of distinction, Pelagian or Oceanic Negroes. I am aware that some objections may be made to this appellation, but I can discover no other name that is on the whole more suitable. The third department of Oceanic nations have been termed Alfourous, Haraforas, and Alforians. To this division the Australian tribes have been referred, and the Alforas have been described as resembling the Australians in the shape of their heads, which display a peculiar type, and in the nature of their hair, which is not crisp or woolly, but straight and long. Of the history of these nations we have very little information on which reliance can be placed. The Australians are the only race included among them whose language is known to be distinct from the Polynesian. As to the various tribes of the islands in the Archipelago referred by voyagers to the Alforian people, it is still undetermined whether they are allied to the Polynesian or Australian stock or constitute a separate family." (Physical History of Man, vol. v, pp. 3-5.)

The name of *Malayo-Polynesian* is given to all those nations of the great Oceanic region whose dialects have been found to bear an affinity to the language of the Malays or people of Malacca. This is well known to be the case with a large proportion of the tribes dispersed through the whole extent of this quarter of the globe; their affinity, which was first suspected from the resemblances of their dialects by Captain Cook and his companions, having been made evident by the subsequent researches of Marsden, Crawfurd, and other learned writers, but more especially by the profound investigations of Baron William von Humboldt. This great stock seems naturally divisible into three branches: the first including the *Indo-Malayan* or *Western Malays* of the Indian Ocean; the second, the *Polynesians* of the Pacific; and the third, the *Madecassians* or inhabitants of Madagascar. A much closer affinity prevails between the idioms of the tribes of the first group, than between their languages and those of the Pacific Ocean. The former, indeed, may be almost said to be dialects of one language; since they are susceptible of analysis by the same grammatical rules, and each contains elements common to all. It is in the Philippine Islands that this common speech displays the fullest and most varied and elaborate development of grammatical forms; but there is reason to regard the same idioms as extending over the whole Indian Archipelago from Sumatra to New Guinea, and as prevailing among the natives of the Ladrones and Caroline Islands, which lie on the borders between the Indian Archipelago and the Polynesian region. The proper Malays are well known as a people of short and slender stature and small limbs, but well-formed and vigorous; they have flat faces, oblique eyes, and features generally resembling the Chinese; they are in complexion considerably darker than that race, as might be expected from their proximity to the equator; but they are much fairer than the Hindoos. They have, in fact, much of the tawny yellow tint of the Northern Asiatics, and resemble them also in the character of the hair, which is black, partially tinged with a deep reddish-brown hue, commonly lank, but sometimes waving in curls. The natives of the Caroline Islands have a decidedly Mongolian countenance; and their complexion is originally of a citron yellow, though it becomes brown by exposure.

The *Polynesian* branch consists of numerous tribes scattered over groups of islands in the Pacific Ocean; and these are for the most part similar in manners and customs to the most rude of the West Malayan islanders, or to those tribes whose simple and primeval state has not been altered by modern habits introduced by the Mahommedan Malays, or by an earlier intercourse with the people of continental India. There is amongst them, however, a much wider diversity, both as to physical and mental characteristics; so that they would not seem, but for their fundamental community of language, to present any near approach to each other, or to the preceding group. This community, however, is so close, as to leave no reasonable doubt of a common origin; for there is no other feasible method of accounting for the fact that the languages of people so remote from each other as those of New Zealand, of Tahiti, of Tonga, and of the Sandwich Islands, should not merely have a fundamental vocabulary common to all, but should have been formed by the same laws of construction. Although their resemblance to the languages of the Malayan branch is less evident than the mutual resemblance of the dialects of the latter, yet it is quite decided enough to place them in one family of languages. The Polynesian tribes are also connected by a general correspondence in their social condition, and in their religious and political institutions.

In regard to physical characters, the Tahitians and the Marquesans may be regarded as the finest specimens of the Polynesian race. They are tall, well made, their figures combining grace and vigour, and their countenances presenting much of the European physiognomy, except in the spreading out of the nostrils, and the thickness of the lips—characters which, though more constant among them, are frequently to be met with amongst individuals of our own race. The skull is for the most part remarkably symmetrical; there is occasionally, however, a slight tendency to the formation of a ridge along the sagittal suture, and to prominence of the upper jaw, as if indicating a slight degradation towards the Negro type. The complexion, especially in the females of higher classes who are sheltered from the wind and sun, is of a clear olive or brunette, such as is common among the nations of central and southern Europe; and the hair, though generally black, is sometimes brown, auburn, or even red or flaxen. The Hawaii or Sandwich islanders stand next to the Tahitians, to whom they are nearly allied in language. Their form and physiognomy are less elevated; their complexion somewhat darker; and their hair, though often soft and flexible, is frequently also crisp and frizzled. The New Zealanders and Ombai islanders constitute the next subdivision. They present great varieties of conformation and complexion; some of them being tall, well-formed, and comparatively fair; whilst others are dark or almost black, and inferior in stature and figure. Yet the language of these people, being a simple Polynesian dialect, gives no indication of the mixed descent between the Polynesian and a supposed dark aboriginal race, which has been imagined by some writers; and they have themselves no tradition which would warrant such an explanation.

Similar varieties in physical character are to be traced among all the other races scattered through the Great Southern Ocean; and if we once admit such a conjectural mode of accounting for them in one case, we must adopt a similar hypothesis in every other, and even this will give no explanation of the appearance of a type so very near the European in several of them. The inhabitants of the Tonga or Friendly Islands, which constitute a fourth group, bear considerable resemblance to the New Zealanders, both in physical characters and in language. They are of stout, vigorous form, less rounded and graceful than the Tahitians, but more muscular. Their features are very various; a truly European cast of countenance, with Roman nose, being frequently to be met with; and few have any peculiar thickness about the lips. The general colour is a cast deeper than the copper brown; but several have a true olive complexion, and some of the women are still fairer. The hair is generally thick, straight, and strong, though bushy and frizzled in a few instances; its general colour is black.

The *Madecassians, Malecassians,* or inhabitants of Madagascar, constitute the third primary branch of the Malayo-Polynesian stock; the chief ground for referring them to it being the affinity of language, which is of the most fundamental character, and not such as could be accounted for by any subsequent intercourse between nations originally distinct. Throughout the whole of this large island only one language is spoken; notwithstanding that the several tribes by which it is inhabited differ remarkably in their physical characters. Much yet remains to be known, however, respecting them. It appears that some among them bear a striking resemblance to the Malayan race; whilst others show a transition to the Negro type in the darkness of the complexion, the thickness of the lips, the breadth of the nose, and the curly and bushy character of the hair; and others, again, approximate to that type still more closely in having black skins and woolly hair. Dr. Prichard thinks that the most feasible method of accounting for these diversities is to suppose that a great part of the Malecassian population was originally derived from Africa, and that the Malayo-Polynesian language was introduced by a colony from the Indian seas, probably from Java, at a time when the Javanese were skilful navigators and formed settlements on various islands of the Indian Archipelago. It is not unlikely, he thinks, that an accurate examination of the different dialects spoken among the black and woolly-haired tribes (which has not yet been made) might display resemblances with the idioms of the Kafirs and the Mozambique Negroes.

The second principal division of the Oceanic races is formed of those black-skinned, ill-favoured savages who are found in the interior of many of the larger islands of the Indian Archipelago, and in some instances constitute their principal or even their entire population. Of these people, termed by Dr. Prichard *Pelagian Negroes,* he gives the following general account, which places them in striking contrast with the Polynesians.

"Ferocious and sullen, of savage and menacing aspect, naturally averse to intercourse with strangers, they have ever shunned the approach of civilized people as uniformly as the Polynesians have courted it. Hence these tribes, their national affinities, and their languages, are still almost wholly unknown, in spite of the diligent inquiries of many intelligent persons who have devoted themselves to the investigation. Their physical characters are likewise very different from those of the agile, graceful, and comparatively fair Polynesians. Among these tribes are to be seen some who recede farthest from the almost European or Asiatic beauty of the Tahitian and Marquesan islanders, and exceed in ugliness the most ill-favoured brood of the African forests, whom they rival in the sooty blackness of their complexion. The natural covering of their head is of various kinds; some have the woolly hair of the Negroes of Guinea; others have lank straight locks, which may be compared with those of the Esquimaux or the Algonquins; while many are striking to the beholder by their broad, bushed-out, and frizzly periwigs, reaching the circumference of three feet, by which they obtained from Dampier the epithet of 'mop-headed Papuas.'" (Physical History, vol. v, p. 213.)

In his 'Natural History of Man' Dr. Prichard had adopted the opinion of Quoy and Gaimard, who, followed by Lesson, maintained that these mop-headed Papuas are a hybrid race, resulting from the admixture of the Malays and the Pelagian Negroes; but he now considers them (on the authority of Mr. Earle, who has had more extensive intercourse with them, and perhaps greater opportunity of correct observation of several of these tribes than any European before possessed) as a pure breed, forming one of the three divisions under which the black races of this part of Oceanica may be arranged. The other two are the puny Negroes of the Indian Seas, which have woolly hair and features resembling those of the Guinea Negroes; and the Australasians and Alforians, which have straight hair and a less Negro physiognomy. To the portion of Oceanica inhabited by these races Dr. Prichard gives the name of *Kelænonesia* or Oceanic Negro-land. Under this designation must be comprised all the insulated countries which lie immediately around Australia, including New Guinea and the whole of Papua-land on the northern side; Tasmania (Van Diemen's Land) on the southern; New Ireland, Solomon's Islands, the New Hebrides, and New Caledonia, on the north-east and east; whilst on the north-west it seems to have originally comprehended the greater part of the Indian Archipelago, extending into the mountainous interior of the Malayan peninsula and to the Andaman Islands in the Bay of Bengal, and northwards to the Philippines. In all these situations, traces of the Pelagian-Negro races remain; but wherever the Malayan races have established themselves, they appear either to have exterminated the Negro tribes, or to have driven them to the mountain fastnesses of the interior. It is in New Guinea, New Britain, New Ireland, Solomon's Islands, New Hebrides, and New Caledonia, that the Pelagian Negroes constitute the chief or sole population. The aborigines of Tasmania (now almost extirpated) appear, from Captain Cook's account of their physical characters, to have belonged to the same group. The inhabitants of the Fijian or Feejeean Archipelago, which lies between Kelænonesia and Polynesia, present us with the remarkable phenomenon of a people speaking a dialect of the Polynesian language, yet distinguished by physical characters bearing a close approximation to the Negro type.

The third and last division of the Oceanic races is that which includes all the native tribes of the great island-continent of Australasia, together with a mountain race in the interior of New Guinea. These alone are properly entitled to the designation of Alfourous or Alforians; the people known by this name, in the Indian islands, according to the recent and searching inquiries of Mr. Earle, being all tribes of the Malayo-Polynesian race who remain in their uncivilized and primitive state. So far as is yet known, there is a general correspondence in physical characters among all the Australian tribes; the differences being only such as may be very reasonably attributed to the scantiness or plenty in the supply of food, operating through many successive generations, according to the districts inhabited by the respective tribes. The following is one of the latest descriptions of them,—that given by Captain Wilkes of the American Exploring Expedition.

"The natives are of middle height, perhaps a little above it; they are slender in make, with long arms and legs. From their wandering life, irregular habits, and bad food, they are extremely meagre; and as their thinness is accompanied by considerable protuberance of the abdomen, it gives to their figure a distorted and singular appearance. The cast of the face is between the African and Malay: the forehead usually narrow and high, the eyes small, black, and deep-set; the nose much depressed at the upper part between the eyes, and widened at the base, which is done in infancy by the mother, the natural shape being of an aquiline form; the cheek-bones are high, the mouth large, and furnished with strong well-set teeth; the chin frequently retreats; the neck is thin and short. Their colour usually approaches chocolate, a deep umber, or reddish black, varying much in shade; and individuals of pure blood are sometimes as light-coloured as Mulattoes. Their most striking distinction is their hair, which is like that of the dark-haired Europeans, although more silky. It is fine, disposed to curl, and gives them a totally different appearance from the African, and also from the Malay and American Indian. Most of them have thick beards and whiskers; and they are more hairy than the whites." (Physical History, vol. v, p. 264.)

It was for a long time supposed that a great variety of languages exists in Australia; every little tribe or horde into which the population is divided being imagined to have an idiom of its own, totally different from the speech of its nearest neighbours. But more extensive and profound researches have had the effect which might be anticipated; showing that there is a much greater coincidence in the several vocabularies than had been imagined; and that there is such evidence of identity in grammatical construction, as to make it obvious that they are all dialects of a common family of languages. Between these languages and the Polynesian dialects, Dr. Prichard points out some curious affinities; but these affinities serve also to connect them with the Tamulian group of languages (derived, as we have seen, from those of High Asia); and the resemblances between the Tamulian and Australian seem to be closer than those which are presented by either to the Polynesian. For example, the particles, which in most languages are prepositions or prefixes, are in the languages of High Asia added to the terminations of words; this character is found also in the Tamulian and Australian languages; but the Polynesian, like many African languages, and like the European, has prefixes and prepositions. These resemblances would lead to the conclusion, which does not seem improbable on other grounds, that the native Australians are an offset from that southern branch of the great Nomadic races of Asia, which very early spread themselves through the Indian and Indo-Chinese peninsula. There is perhaps no nation, except the Bushmen and the most degraded of the Negroes, whose physical condition is so miserable, or whose mental state appears so low; yet the statements of recent observers, especially of Captain Gray, indicate that the usual ideas on this point are greatly exaggerated.

Notwithstanding the degradation of their physical condition, and the want of success hitherto experienced by those who have made attempts at Christianizing them, there is no reason whatever to believe that the Australian aborigines are destitute of any of the intellectual or moral attributes which are possessed by the highest races of men. The remark has been frequently made by those who have adequate opportunities of observation, that Australian children are in general quite equal to English children in their manifestations of intelligence and capacity for acquiring knowledge; and although their mental development may earlier cease (as seems to be the case with the Negro races), so that they remain, as it were, great children all their lives, yet there is every reason to believe that the education of a few generations would produce the same kind of improvement in them as it has done in other races previously uncultivated. Although they appear to have less susceptibility than many other rude nations to religious impressions, yet it is quite certain that they are not destitute (as some have represented them to be) of all idea of a God: they seem to have a notion, also, of a future state, and believe in good and evil angels. They have likewise a superstitious belief in magicians or sorcerers; another point of connexion with the nations of High Asia. It is remarkable that in many of their most complex and singular institutions, they present a very peculiar resemblance to many tribes of North American Indians.

The almost total ignorance which at present prevails in regard to the languages of the other black races of Kelænonesia, prevents us from being able to determine their probable relationship to the Australians; but at present it would seem proper (in the absence of any sufficient evidence to the contrary) to associate them all together, and to regard them as the primitive inhabitants of this extensive region, including nearly the whole of the Indian Archipelago with Australia. The relations of the Australian languages to those of High Asia seem to confirm the idea which the geographical position of this region would suggest,—namely, that these races originated from the great stock of Central Asia; coming off from its southern or south-eastern extension at a very early period, before language and civilization had made any great progress. At a later time a second colonization took place,—that of the Malays; who seem pretty certainly to be an offset from some of the nations, now more elevated in civilization and more fixed in physical characters, of the Indo-Chinese peninsula. These people spread themselves through the Indian Archipelago; but their tendency was rather eastwards than southwards; so that instead of possessing themselves of New Guinea and Australia, they gradually migrated through the islands of the Polynesian region. There may seem more difficulty in accounting for the peopling of those remote islands from any one source, than there is in understanding the spread of the human race over the whole remainder of the globe. But the difficulty is more apparent than real; for not to mention the analogous phenomena in regard to the introduction of plants and animals from distant regions, which the creation of new islands by the growth of coral is continually presenting to us, there is evidence that the accidental transport of parties of the islanders to lands very distant from their own is of no very unfrequent occurrence. Several cases of this kind are collected by Mr. Lyell, from the accounts of Cook, Forster, Kotzebue, and Beechey, within whose own knowledge they occurred; and these would of course bear no comparison to the actual number of such occurrences. In the instance mentioned by Kotzebue, a party which was passing from one island to another in a sailing boat was driven out of sight of land, and remained at sea for eight months, during which time they supported themselves by fishing. They at last drifted to an island *fifteen hundred miles* from the one they had left.

If the Malayo-Polynesian nations are to be regarded as really the offspring of one family, we find in the ramifications of that stock examples of almost every variety of the human species. Thus the proper Malayan tribe differs little in its physical characters from the Indo-Chinese type; but among the well-fed Polynesian islanders, the stature and fulness of the body have increased, and the form of the skull and the character of the physiognomy have come to approximate those of the Hindoo or European. In many of these islands, the prevalent complexion among the mass of the people, who are continually exposed to the agency of the climate, is much darker than that of the genuine Malays; the hair becomes somewhat crisp, and the features ugly, presenting a decided approach to the characters of the Pelagian Negroes; and yet among the very same people, the superior caste, who pass their days in luxurious ease, and have little occasion to expose themselves to the sun, have a fair complexion and all the characters of a highly developed or xanthous constitution. This contrast is very remarkable in the Tahitians and Marians.

"It seems to have been the ultimate and full persuasion of all those persons who have made a long abode in the islands of the Pacific under circumstances favorable to accurate investigation, that these phenomena can only be explained on the supposition that they result from the agency of climate and physical influences on the original race. The appearance of a xanthous complexion under moderate temperature and among people living in a state of protection from severities of climate is so common an observation, and one that we have already traced in so many instances in almost every other part of the world, that we may well look for it in the Polynesian Islands; and there, when we find this change connected and coextensive with another physical change, we may fairly infer that these connected phenomena have one and the same cause. I allude to changes in the stature, the form of the head, the quality of the hair, &c. There seems to be no other hypothesis, if we open the widest field to conjecture, that can in any way explain all the phenomena of physical variety that display themselves in the Oceanic region, and this sufficiently accounts for all of them,—namely, the deviation of the primitive Malayan or Indo-Chinese type on the one side to the character of the European, and on the other to a conformation of body very similar to that of the African." (Physical History of Mankind, vol. v, p. 285.)

The aboriginal people of *America* are generally considered as a department of the human family very distinct from the inhabitants of the Old World; and attempts have been frequently made to define the race by physical characters. But these attempts have been founded upon a very limited acquaintance with the nations of this vast continent; for, taken in the aggregate, they are by no means uniform either in physical or moral qualities; nor is the line of distinction between them and the rest of mankind so obvious or strongly marked as is usually imagined. Thus the native Americans have been described as "red men;" but there are tribes equally red, and perhaps more deserving that epithet, in Africa and Polynesia; and the American nations are by no means all of a red or coppery hue, some being as fair as many European people, others being brown or yellow, and others nearly if not quite as black as the Negroes of

Africa. Again, it has been attempted by anatomists to distinguish the American races by a certain configuration of skull and form of features; and even Dr. Morton, in his splendid 'Crania Americana' (noticed by us in our tenth volume), has given his authority in support of the opinion that such distinctive characters existed "in the squared or rounded head, the flattened and vertical occiput, the high cheek-bones, the ponderous maxillæ, the large quadrangular orbits, and the low receding forehead." Nevertheless he is obliged to admit that very considerable diversities present themselves in cranial conformation; and he altogether excludes the Esquimaux, who, according to the evidence of language, must be regarded as being as truly an American people as any other, in spite of the manifest approach which they present to the Northern Asiatic type of cranial configuration. The testimony of those who have actually travelled in different parts of America, and who have scrutinized with observant eyes the physiognomy and form of head, not in individuals only but in nations, must be admitted to be much more satisfactory; and amongst these that of the distinguished naturalist, M. d'Orbigny, must be regarded as especially worthy of attention. "As a general position," he says, "we may regard each particular nation as having between its members a family resemblance, which, distinguishing it clearly from its neighbours, permits the practised eye of the zoologist to recognise in the great assemblage of nations all the existing types, almost without ever confounding them. *A Peruvian is more different from a Patagonian, and a Patagonian from a Guarani, than is a Greek from an Ethiopian or a Mongolian.*" Nor will any epithet derived from their habits of life apply to all the tribes of this quarter of the globe. The native Americans are not all hunters; there are many fishing tribes among them; some are nomadic, others cultivate the earth and live in settled habitations; and of these, a part were agriculturists before the arrival of the Europeans, whilst others have learned of their conquerors to till the soil, and have changed the ancient habits of their race, which, as we may hence infer, were not the necessary result of organization or of fixed congenital propensity.

Yet it will be found on close examination that certain characters are discoverable, which are common, or nearly so, to the whole of this group of nations; that there are strong indications, if not proofs, of a community of origin or of very ancient relationship between them; and that, in surveying collectively the people of the New World, we are still contemplating the same bodily and mental constitution as that which we have seen to prevail among the people of the Old, although it presents itself, in many respects, under a peculiar aspect. When we attempt, however, to ascertain their family relationship to the races of the eastern hemisphere, we find every indication tending to prove the antiquity of their separate existence; so that we must regard them as having constituted a distinct department or family of nations from the first dispersion of the human race. It is not to be expected, therefore, that we should be able to discover proofs of their derivation from any particular tribe or nation of the Old Continent; though, as we shall hereafter see, there are not wanting indications of their community of descent with certain races of the latter.

The most decided and clearly marked evidence of mutual relationship between the American nations is to be found in the characteristic *structure* of their languages. On this subject we have enlarged sufficiently in our former article, (pp. 53-55); and we need not, therefore, dwell further on it here, except to remark, that although this mutual relationship has been found in all the American languages which *have been* examined, including the most remarkable dialects in very distant parts of America, yet that many are still altogether unknown; so that the evidence cannot yet be regarded as complete. It is a very curious circumstance, and one which cannot be regarded as a mere accidental coincidence, that the language of the Old Continent to which the American languages bear the nearest resemblance in their most characteristic feature, that of the formation of words by the process of *agglutination*, should be the Euskarian or Basque. Upon this point we shall have more to say presently.

There are, besides, many remarkable traits in the moral and social state of the American nations, which indicate some near relationship amongst them, and serve to distinguish them from the races of the Old World. Attentive observers have been struck with the manifestations of greater energy and mental vigour, of more intense and deeper feeling, of a more reflective mind, of greater fortitude, and of more consistent perseverance in their various pursuits and enterprises, when they have compared the natives of the New World with the sensual and volatile, and almost animalized savages, who are still to be found in some quarters of the Old Continent. They have been equally impressed by the sullen and unsocial character, by the proud apathetic endurance, by the feeble influence of the social affections, by the intensity of hatred and revenge, and by the deep malice-concealing dissimulation, so remarkable in the dwellers amid the dark solitudes of the American forests. Among many of the American nations, moreover, traces have been observed of ancient institutions, of a nature that seems to imply the existence of much refinement and of an artificial state of society. Of this description are,—a complicated form of government, regulated despotisms or monarchies, privileged orders, hierarchical and sacerdotal ordinances, systematic laws (the results of reflection and a settled purpose) connected with marriage, inheritance, and family relationships, and other customs, all of which are strongly contrasted with the simple and unreflective habits of rude and uncivilized nations. Their opinions, moreover, respecting a future state, and the nature and attributes of invisible agents, are strikingly different from those of nations who have never emerged from primitive barbarism. They have had in use, too, from time immemorial, cultivated plants and domesticated animals, different from those of the Old World; and their earliest traditions refer the knowledge of these to some fabulous person who descended from the gods, or who suddenly made his appearance among their ancestors; thus indicating the remoteness of the era of their separation from the inhabitants of the Old World, who have similar mythical legends in regard to the introducers of *their* first arts and acquirements.

Abundant evidence is afforded, by architectural and other remains still existing, as well as by the accounts of the early Spanish historians, of the high degree of civilization which some of the nations of America had formerly attained, especially in the warmer regions of that continent. Thus the natives of Mexico erected stupendous edifices, which rivalled those of Egypt; and although they could not yet attain to the greatest of human inventions,—that of symbols representing the sounds of words,—they had long aspired after it, and had contrived a method of recording events and of handing down to memory the passages of their ancient history. They had even made great advances in science, and had a solar year with intercalations on the principle of the Roman calendar. They were diligent cultivators of the ground, and also expert miners and workers in metals; even astonishing the workmen of Europe with the skilful manner in which they set gems. They appear, too, to have been influenced by a deep sentiment of religion, and had a very stately and majestic ceremonial. Nevertheless they do not seem to have derived from these advances in civilization any moral improvement, or any mitigation of that sullen malignity which seems common to the native tribes of the New World; and their religion was far from having an exalting influence, since their gods had no attribute of clemency or mercy, but were invested with the worst forms of their own dark passions. In Peru, also, we find remains of Cyclopean structures erected during the government of the Incas, which bear comparison with those of ancient Egypt; and the wonder is increased when it is recollected that no beast of burden save the llama existed in Peru before the Spanish invasion. At a time when no public highways existed in England but such as were relics of Roman greatness, there were roads of 1500 miles in length in the empire of Peru, carried over heights which overtop the Peak of Teneriffe. The ancient Peruvians were ignorant of the manner of forming an arch; but they had constructed suspension bridges over frightful ravines. They had no implements of iron; but they could move blocks of stone as huge as the Sphinxes and Memnons of Egypt.

Among the peculiar customs of the American races, is that of altering the form of the skull by artificial compression, which may be traced in different parts, both of the northern and southern divisions of the continent. For an account of this custom, and of its effects, as given by Dr. Morton, we must refer to our former notice of his work. (Vol. X, p. 478.)

We cannot venture upon any survey of the individual nations of the American group, but must confine ourselves to a general summary of the results of such an investigation. In the first place, then, whether it be supposed that they have originated in an offset of the Asiatic races, or that they have had a distinct parentage, it must be admitted that, if the American nations have had a common ancestry, most of them have subsequently departed widely from the original type; and no other reasonable cause can be assigned for such departures, than the tendency to variation inherent in the human constitution, especially under the influence of a change in external conditions. Secondly, if this modifying influence be *not* admitted, but it be still maintained that the characters of the several races of mankind are fixed and unalterable except by mutual admixture, we must allow not *one* only, but *many* distinct progenitors for the several groups of American nations that present well-marked diversities from each other; a supposition altogether improbable, especially when the mutual affinities of their languages are duly considered. Thirdly, as the modifying influence of external circumstances, once admitted, enables us to account for the descent of the American races from an Old World stock, as readily as from a parentage peculiar to themselves, there is no *a priori* improbability in the way of the supposition that the American continent was first peopled from the Asiatic. Fourthly, the strongly-marked resemblance presented by the Esquimaux to the Northern Asiatics in physical characters, whilst their language is decidedly American, seems to indicate that they form the connecting link between the two great races.

"There seems to be no great difficulty in the supposition that people from some tribe or tribes of the extreme north-east crossed over Behring's Strait, or passed along the Aleutian chain of islands from Asia to America in an early period, and formed a nation in the New World, who, after constructing or rather developing into its complex form a primitive speech, of which all the polysynthetic idioms are derivatives or imitations, spread themselves over the whole continent of America, and being thereby scattered, soon separated into particular hordes, which became the germs of many particular nations. The number and diversity of languages at the present day require that we should assume an early era for this event; and the rapidity with which the human species is propagated under favorable circumstances removes all difficulty that may attend the supposition." (Physical History of Mankind, vol. v, p. 546.)

The people who thus colonized America would doubtless be some of the northern Asiatic tribes who braved the cold of the Icy Sea, and supported themselves chiefly by fishing. The Esquimaux constitute just such another race; and may not improbably be regarded as the nearest representatives of the ancestral type, from which the offsets that have migrated in a southerly direction have long since departed. Thus, we see a coincidence between the geographical probability (if we may use the expression)—that is, the probability that the first American colonization took place from some northern Asiatic nation in consequence of the comparative facility of access from this part of the Old Continent,—and the correspondence in physical characters between the most adjacent of the Asiatic and American races, which strongly supports the idea of their close family relationship; and although the entire difference between the American and Asiatic languages might at first sight appear to be a powerful argument on the contrary side, yet the reappearance of the most universal and characteristic feature of the former, in what there is strong evidence for regarding as a remote offset from the latter,—namely, the Basque or Euskarian language,—must be admitted as an argument of no inconsiderable weight in the other direction. On this supposition, the original languages of High Asia contained an element which has never been manifested in themselves, but which has been partly developed in one of the early offsets of that race, the Iberian nation, and still more fully in the American, which was probably the result of a still earlier migration. Nor should the strong resemblance which has been pointed out between the peculiar customs and institutions of the Australian savages and the North American Indians be without its influence on our decision; for if *other* facts tend to indicate the common origin of these races—remote and almost antipodal as they now are,—the coincidence must be regarded as a confirmation of that supposition, not to be lightly set aside.

Thus, then, the most careful and extensive researches into the physical history of mankind tend to break down the barriers which ignorance and prejudice have erected between its different races: by showing that there is nothing more improbable in the supposition of their common origin in a single pair, than in three, four, five, or any small number of contemporaneous ancestors; since in each case the modifying influence of external circumstances must alike be admitted to account for the marked diversities that can be shown to exist amongst the existing races whose origin would be referred to a common ancestry. And the supposition that each race at present distinguishable by well-marked physical characters had an originally distinct parentage, would lead to such a multiplication of primary stocks as we fancy that none of the advocates for such a doctrine are prepared for; besides being inconsistent with all the evidence of common descent, which is furnished by physiological, psychological, and philological considerations. In Dr. Prichard's first volume will be found much interesting evidence in favour of the specific unity of the human races, derived from their conformity in physiological characters,—such as the average duration of life, the periodical phenomena of the constitution, the phenomena of hybridity, and the liability to the same diseases; the chief points of which were adverted to by us, at a very early period of our critical labours, soon after the publication of that volume (see Vol. III, pp. 365 et seq.) He has also brought together a most interesting mass of evidence of identity in psychical nature between the highest or most civilized and the lowest or most degraded nations; which, in his shorter work, he thus sums up:

"We contemplate among all the diversified tribes who are endowed with reason and speech, the same internal feelings, appetencies, aversions; the same inward convictions, the same sentiments of subjection to invisible powers, and (more or less fully developed) of accountableness or responsibility to unseen avengers of wrong and agents of retributive justice, from whose tribunal men cannot even by death escape. We find everywhere the same susceptibility, though not always in the same degree of forwardness or ripeness of improvement, of admitting the cultivation of these universal endowments, of opening the eyes of the mind to the more clear and luminous views which Christianity unfolds, of becoming moulded to the institutions of religion and of civilized life: in a word the same inward and moral nature is to be recognised in all the races of men." (Natural History of Man, p. 546.)

The philological evidence in favour of the specific unity of the human races is to be found not merely in the coincidence of particular features of the languages of remote families of nations, but also in the more general fact of the universality of spoken language, and in the power of translating from one language to another. Dogs and monkeys *may* have languages of their own; but there is no such relation between either of them to ours as may enable us to become acquainted with them; and where brute animals have been taught to comprehend human language, it has been that only which related to certain objects well known to them with which the sounds of the words could readily be associated. Let this limited comprehension be compared with that which is manifested by such human beings as seem most completely cut off, by the deficiency of organs of sensation, from communication with their fellows, and the contrast is at once evident. The lighting up of the whole countenance of Laura Bridgman, which her benevolent and persevering instructor, Dr. Howe, describes as having taken place when she first made the discovery that she could express her thoughts in words by a combination of literal signs, and the gradual elevation of her whole mind through the medium of language, from the level (but little above that of the brute) in which it would have probably ever remained if she could not be made to comprehend its use, afford most striking testimony to the fundamental importance of this common characteristic of humanity. The power of fully rendering the thoughts conveyed in one language into another tongue, must of course depend in great part upon the relative advancement of the two languages. The profound ideas of a German philosopher could not be expressed in the Hottentot gibberish; nor does the peculiar style of eloquence cultivated in the East produce its adequate effect when rendered in our own language. But any two barbarous languages, or any two which are highly cultivated, are so pervaded by a sameness of character as to bear witness to the identity of their internal source.

Contenting himself with having established to his satisfaction the main positions of the common origin of the human races, Dr. Prichard does not offer any speculations in regard to their primary source. Perhaps he considers it safer not to give the weight of his authority to any such speculations, until they may have a better foundation than that on which they can be at present built. But as we are not restrained by any such consideration, we shall briefly state the idea which had occurred to our own minds, and which has gained strength by the more detailed survey which we have been recently led to make (for the purposes of this review) of the vast collection of materials brought together by Dr. Prichard.

The stock from which the globe was originally peopled appears to us to be most nearly represented at the present time by the people of High Asia, namely, the Mongolians and their allies; and some part of that region was probably their original seat. We ground this hypothesis (for we wish it to be regarded as nothing else) upon the following considerations. The physical characters of these people are such as peculiarly adapt them to a nomadic life; they have a vigour of constitution which enables them to brave great diversities of climate; and they readily accommodate themselves to a great variety of hardships. Their natural disposition leads them almost to seek these rather than to avoid them; and to wander over the surface of the earth, rather than to settle in any one spot. If it had been intended that by such migrations the whole globe should be peopled, no better *centre* could be thought of than Higher Asia; whose connexions with all other lands are such as are possessed by no other region; and whose climate is so intermediate between that of the frigid and the torrid zones, that the passage into either may be effected without any violent transition. This *a priori* argument, however, would be worth but little, if we did not find it in correspondence with the very curious fact, that the most ancient inhabitants of almost every part of the globe are connected with the nations of High Asia, more or less closely, by affinities of language or of physical characters. This we have seen to be the case, not merely with the aboriginal people of Northern and Southern Asia, but with those of Northern and Southern Europe, with those of the Caucasus, with the first settlers in the Indian Archipelago, and (as seems highly probable, though the proof is less complete) with the American nations. The only region regarding which there is not the same decided evidence is Africa; but until the contrary shall have been shown, we cannot but think that the existence of a people presenting so strongly the original Mongolian characters (modified, however, by change of climate, &c.) as do the Hottentots, affords strong evidence that this continent also was early peopled from the same stock. There is ample evidence that the Mongolian type is capable of being elevated on the one hand towards the Indo-European, or degraded on the other to the Negro or Australian; and we are not aware that either of the other types exhibit the same capacity for metamorphosis.

In certain spots of the globe thus peopled with races derived from a common centre, varieties in physical conformation sprang up; new and more refined languages were originated; and subordinate centres were thus formed, from which more limited radiations have subsequently taken place, impressing their own features of civilization upon the countries through which they have spread. Thus we have, at a very early period, indications of the Egyptian, the Syro-Arabian, the Indian, the Indo-Chinese, the Mexican, and the Peruvian races; and although some of these may possibly have been mutually connected at their origin (as the Egyptian and the Indian), they seem to have been very early separated, and to have attained their fullest development quite independently of one another. Each of these had its own peculiar characters, departing more or less from the common original; and contained within itself those elements of progress, of fixity, or of early decay, which have exerted their influence upon the subsequent destiny of these races. The great body of African nations is the one whose origin has seemed to us most obscure; but the recent investigations which have been made in regard to the languages of some of their most elevated tribes, would lead us to regard this continent as having been peopled (after its first and most scattered colonization) by successive offsets from the stem which produced the Syro-Arabian races. The earliest of these, which wandered the farthest from the original centre, would seem to have most completely lost the traces of its original ancestry, both in respect to language and physical characters; whilst the later colonies, which have not extended themselves beyond the northern and eastern portions of the continent, have retained more or less of the primitive type of language, and usually show less approximation to the true Negro physiognomy. Nevertheless the Syro-Arabian variety does exhibit a greater tendency to this kind of change than we know to be the attribute of any other race; since instances are by no means wanting, in which, under the prolonged influence of similar external conditions, families and whole tribes of undoubted Arab descent have assumed all the most important characters of the Negro races.

It has been remarked by an acute critic, that the lapse of time requisite to bring about such changes as those required in any hypothesis of the single origin of the human races, is far greater than the limits of received history allow. This remark is commented on by Dr. Prichard in a supplementary note at the close of his last volume; which is not one of the least interesting parts of his work. He points out that all those writers who have entered upon the investigation of primeval history, have felt a difficulty in reconciling the proofs of the early existence of powerful empires and high grades of civilization with the ordinary chronology founded upon the Mosaic records; even though not at all disposed to impugn the authority of the latter. And then, in a truly philosophical but not less reverential spirit, he proceeds to inquire how far that chronology is necessarily to be regarded as complete and authentic, by those who receive the narrative as the genuine record of Divine Revelation. He comes to the conclusion which might be expected from so learned and judicious a critic, —that the Book of Genesis was not intended to give us an exact chronology, any more than it was intended to teach us geology or astronomy; that it is a collection of different documents having no proper connexion with each other,—that the genealogies which it includes have no claim to completeness,—that the great length of lives recorded in the ante-Abrahamic times is founded on a mistake in the interpretation of numbers or numerical signs,—and that there is consequently no foundation whatever for a chronology of the earliest ages, or, in other words, that no means are to be found for ascertaining the real age of the human race. Consequently we need not be fettered by any considerations of this kind in our attempts to determine the mode in which the globe became peopled by the nations, which are now spread over every part of its surface that is fit for the habitation of man.

It now only remains for us to place before our readers a general estimate of the merits of Dr. Prichard's ethnographical labours; and we cannot do so more concisely or pointedly, than by adopting the language of a learned contemporary, to whom we have more than once had occasion to refer, and whose judgment, in this particular, we would most entirely and cordially adopt as our own. Speaking of the 'Physical History,' he says,—"It is impossible to even turn through the pages of Dr. Prichard without admiring the very varied erudition brought together for the illustration of one topic. The knowledge of a modern physiologist is in itself sufficiently multifarious. Blumenbach and some others had so far led the way upon this question, as to have preoccupied all the main physiological discussions and the most prominent facts concerning human races. But Prichard has not merely filled out with an almost inexhaustible learning the topics previously familiar, and endowed with new force arguments which had been almost abandoned as untenable; he has further investigated ancient and modern races with a detail perhaps previously unattempted, scraping together and illustrating with great sagacity all the scattered notices found in Greek or Roman writers, and combining for his purpose the ethnographical information furnished by modern travellers, missionaries, and philologists. The latest researches of acute and inquisitive Germans, whether concerning the inscriptions of ancient Italy, the characters of Persepolis, or the language of Biscay and India, with the splendid geographical generalizations of Karl Ritter, are all made to converge into the same focus. The value of the work before us by no means depends upon the question whether the writer has or has not proved, either that man is one species, or that this species descends from one pair. It is, in fact, a storehouse of information concerning the whole controversy. The adverse arguments, although not pressed and developed as by an advocate, are set forth without disguise or suppression, so far as we are aware; so that in no small degree the reader is put in possession of materials for an independent judgment. Add to this,—what is in our view of peculiar importance, quite independently of the great cause which the book is pleading—the facts themselves, so varied, so instructive, so picturesque, and so very eloquently set forth, are of high interest; and the learning, ancient and modern, crowded into its pages, is such as would with difficulty be gleaned from ample libraries by the most persevering study. The work, in short, has been the labour of a life. Thirty-seven years have elapsed since the accomplished author laid its foundations, and three times has it been reconstructed."* As a striking contrast with the hasty manner in which some other writers have been accustomed to dogmatise on the subject, we may recall the attention of our readers to the fact, that the first volume of this third edition was published as much as eleven years since, soon after the commencement of our critical labours. That the delay in its completion has been owing to nothing else than the author's determination to render it as perfect as possible we can vouch for, from our own knowledge of the unremitting devotion of his disposable time to his laborious investigations. We rejoice that we are enabled, at the close of our own career, to congratulate him on the successful attainment of this step in his honorable course. That he will not consider it as here concluded, but will be constantly availing himself of every kind of information that may throw light upon the obscurer departments of Ethnology, all who have the privilege of a personal knowledge of him must feel the utmost confidence.

In regard to the smaller work it will be sufficient to say, that its object is to furnish for the use of general readers a brief and popular view of all the physical characteristics, and likewise of the moral and intellectual peculiarities which distinguish from each other the different races of men; and likewise to offer such an account of the nature and causes of these phenomena as the present state of our knowledge will afford. It may, therefore, be regarded as a popular abridgment of the larger work; comprising the chief results of the inquiries pursued in the latter, with a general outline of the evidence on which they are based: the 'Physical History' is the work for the man of science; the 'Natural History' for the general reader.

---

### Art. XV.

* New Quarterly Review, vol. viii, p. 98.

